# Dynamics of thymol dietary supplementation in quail (*Coturnix japonica*): Linking bioavailability, effects on egg yolk total fatty acids and performance traits

**DOI:** 10.1371/journal.pone.0216623

**Published:** 2019-05-09

**Authors:** Maria E. Fernandez, Jackelyn M. Kembro, Maria L. Ballesteros, Jorge M. Caliva, Raul H. Marin, Maria C. Labaque

**Affiliations:** 1 Consejo Nacional de Investigaciones Científicas y Técnicas (CONICET), Instituto de Investigaciones Biológicas y Tecnológicas (IIByT), Córdoba, Argentina; 2 Universidad Nacional de Córdoba, Facultad de Ciencias Exactas, Físicas y Naturales, Instituto de Ciencia y Tecnología de los Alimentos (ICTA), Córdoba, Argentina; 3 Universidad Nacional de Córdoba, Facultad de Ciencias Exactas, Físicas y Naturales, Cátedra de Química Biológica, Córdoba, Argentina; 4 Consejo Nacional de Investigaciones Científicas y Técnicas (CONICET), Instituto de Diversidad y Ecología Animal (IDEA), Córdoba, Argentina; 5 Universidad Nacional de Córdoba, Facultad de Ciencias Exactas, Físicas y Naturales, Cátedra de Diversidad Animal II, Córdoba, Argentina; 6 Universidad Nacional de Córdoba, Facultad de Ciencias Exactas, Físicas y Naturales, Cátedra de Ecología, Córdoba, Argentina; University of Illinois, UNITED STATES

## Abstract

Phytogenic additives such as thymol are encountering growing interest in the poultry industry. However, there are still questions concerning dynamics of their bioavailability, biological action, optimal dosage and duration of supplementation needed to achieve meaningful effects, as well as persistence of induced changes after supplement withdrawal. We studied the link between the dynamics of free thymol concentration and the changes in fatty acids composition in quail egg yolk, both during a month-long chronic dietary supplementation and after 3 weeks of supplement withdrawal (post-supplementation). Fifty, 85 days-old, female quail of homogeneous body weights (251±1g) in egg-laying peak were used. To evaluate potential dose-dependent effects, three increasing doses 2, 4, and 6.25 g of thymol/kg of feed (THY2, THY4 and THY6, respectively) and two controls were evaluated (n = 10). In parallel, we assessed the concomitant changes in free thymol excretion, potential liver histopathological changes, and birds´ performance traits. Egg yolk and droppings show a dose-dependent increase in THY concentration after 9 days of supplementation and a decrease after post-supplementation. In egg yolk, these changes were accompanied by reduced saturated fatty acid concentrations achieved by 28 days of supplementation in THY2 and 14 days of supplementation in THY4 and THY6. However, after post-supplementation the aforementioned effect disappeared in THY2 but not in THY4 and THY6. While THY2 failed to increase polyunsaturated fatty acids, THY4 and THY6 increased polyunsaturated fatty acids by day 14 of supplementation and remained increased after post-supplementation. Fatty acids changes induced by thymol are consistent with improved nutritional quality of eggs. No treatment effects were observed in liver histopathology and female performance. Findings suggest that both dose of thymol and duration of supplementation modulate thymol and fatty acids concentrations in egg yolk and thymol concentration in droppings. Furthermore, the persistence of those effects after post-supplementation period is also a dose-dependent phenomenon.

## Introduction

Phytogenic additives are encountering growing interest in poultry nutrition due to their potential beneficial influence on lipid metabolism, useful gut microflora, nutrient absorption, performance, health and welfare [1–3]. In particular, dietary supplementation with thymol (THY; 2 isopropyl-5-methylphenol) or mixtures that contain THY, has been proposed as strategy to enhance poultry productivity in parameters such as growth, feed conversion, egg laying rate and egg physico-chemical quality [4–7]. Additionally, THY potential for modulating the fatty acid (FA) profile and preventing lipid peroxidation in certain tissues of a variety of species [8–10] has promoted research aimed at improving nutritional quality of meat [11–13] and egg products [14–16]. From a human nutritional standpoint, consuming products with higher levels of n-3 polyunsaturated FA (n-3 PUFA) and lower saturated FA (SFA) is associated with a number of physiological and health-beneficial effects, such as prevention of some chronic diseases [17,18]. Also, in the context of early avian nutrition, adequate amounts of PUFA in the yolk are essential to meet the demands of the developing embryo [19,20]. Modulation of the FA profile by diverse dietary supplements containing THY has been largely attributed to THY antioxidant activity via scavenging of free radicals, enhancing the bird’s endogenous enzymatic and non-enzymatic antioxidants, chelating of metal ions and regulation of different signaling pathways [5,6].

As a bioactive compound, THY has potential for physiological effects at various levels [21]. *In vitro* results in different cell lines and experimental systems showed that THY promotes dose-response effects (reviewed in [6]). However, *in vivo* studies in poultry, reflect inconsistencies in relation to dose-response effects, which can be explained, in part, by substantial differences in supplementation protocols used [4,13,22–26]. In particular, there are fundamental differences regarding presentation form of the supplement (ranging from herbs to commercial mixtures with several EO), doses administered (<mg to g/kg feed), duration of supplementation (days to months) and age of animals supplemented (chicks to adults), which could ultimately impact, among other aspects, on bioavailability or amount of the supplement that can be absorbed, used and/or stored by the animal [21,27,28]. Although the contributions of these studies are of great importance, the wide range of supplementation protocols hinders an adequate understanding of the scope of THY effects, as well as the characteristics of its *in vivo* mode of action, thus, making it difficult to identify supplementation aspects (e.g. absorption, target tissues, etc.) that are important in order to promote accurate nutritional recommendations. This background highlights the need for studies using pure THY (as a single compound), at various doses throughout the supplementation protocol, to determine whether there are doses or durations of supplementation thresholds capable of promoting biologically relevant responses.

In species such as rabbits, pigs, rodents and humans there is a fairly good number of studies on THY absorption, digestion, metabolism, excretion and bioavailability, which provide valuable background information in the context of the nutrition of monogastric animals in general. However, these studies have been conducted in acute administration and provided short-term data regarding the permanence of these compounds and/or their derivatives in the animal body [29–33]. On the contrary, as stated previously, application protocols proposed in animal nutrition involve chronic supplementation of these compounds. In this regard, few studies in poultry evaluated the bioavailability or concentration of supplemented compounds in target tissues. The scarceness of this information has been attributed partially to methodological difficulties. Recently, methodologies based on HS-SPME coupled to GC-MS and SPE coupled to UHPLC-QTOF-MS have been developed, enabling these determinations [34–37]. Of special interest for us are the results in broiler chickens [34] demonstrating that THY from thyme herb (consisting of leaves and flowers without stems) at 0, 1, 2, 3 and 10 g/kg of feed is efficiently absorbed, distributed to various tissues and excreted after a supplementation trial of 35 days. They also observed an increased intestinal THY concentrations in the group with 10g/kg feed compared to the other groups. Likewise, it has been demonstrated that THY concentration in the egg yolk reached a plateau after 12 days of dietary supplementation, in laying hens fed with thyme extracts at doses equivalent to 2.24 and 3.36 g of THY/kg feed [38]. Additionally, they observed that once supplementation was withdrawn, THY concentration of the yolk decreased gradually. However, these studies, as mentioned above, not only use supplements that are mixtures of different compounds, but also they lack a joint assessment of the dynamics of changes in bioavailability and biological effects (i.e., feed intake, body weight, egg physico-chemical characteristics, etc.) potentially promoted during supplementation (S) or in the post-supplementation (pS) period. This approach is key to understand how supplemented compounds modulate beneficial effects on animal body, thus contributing to determine to what extent the biological effects depend on the circulating levels and/or the concentration of the active compounds in target tissues [21,28]. Measuring bioavailability may also be relevant in context of the safety of edible animal by-products (i.e., eggs and meat).

Although THY has the ‘generally recognized as safe’ (GRAS) status of US government-approved food additives, with safety levels calculated in a wide range of animal species by the European Food Safety Authority [39], further studies complementing toxicological and persistence effects after chronic supplementation *in vivo* are still needed. Moreover, recent reviews have also call for the need of detailed assessment of toxic effects on liver, due to its role in phenolic compounds' metabolism, as xenobiotic biotransformer and in circulatory system [5].

Thus, we studied the link between the dynamics of free THY concentration and the changes in FA composition in quail egg yolk, both during a month-long chronic dietary supplementation and after 3 weeks of supplement withdrawal. To evaluate whether the observed effects are dose dependent, 3 increasing doses of THY (2, 4, and 6.25 g of THY/kg of feed) were evaluated. In parallel, we assessed the concomitant changes in free THY excretion as a non-invasive indicator of the presence of THY in circulation. We also monitored potential liver histopathological changes due to toxicity, as well as female performance traits.

## Materials and methods

### Ethical statement

All experimental procedures were in compliance with the *Guide for the Care and Use of Laboratory Animals* issued by the National Institute of Health [40]. The experimental protocol was approved by the Institutional Committee for the Care and Use of Laboratory Animals (Comité Institucional para el Cuidado y Uso de Animales de Laboratorio (CICUAL) of the Facultad de Ciencias Exactas, Físicas y Naturales, Universidad Nacional de Córdoba (ACTA 4/2015 Resolución 571-HCD-2014). The raw data of all variables presented herein is publicly available on figshare, doi: 10.6084/m9.figshare.7589066.

### Animals and husbandry

Adult female Japanese quail (*Coturnix japonica*) taken from a population of a single 230-bird, hatched in our laboratory, were used in this study. Husbandry was performed according to laboratory routines described elsewhere [16,41]. Briefly, at 28 days of age, females were individually housed in cages measuring 20cm × 45cm × 25cm (length × width × height), allowing them to establish visual and auditory contact with each other, while permitting individual measurements of feed consumption and egg production. An individual feeder and an automatic nipple drinker were positioned in each cage. At all stages feed and water were provided *ad libitum*. The photoperiod was 14h light and 10h dark (0600–2000h; approximately 300–320 lx). The environmental temperature was maintained at 24 ± 2°C.

### General procedure

**Fig 1:**
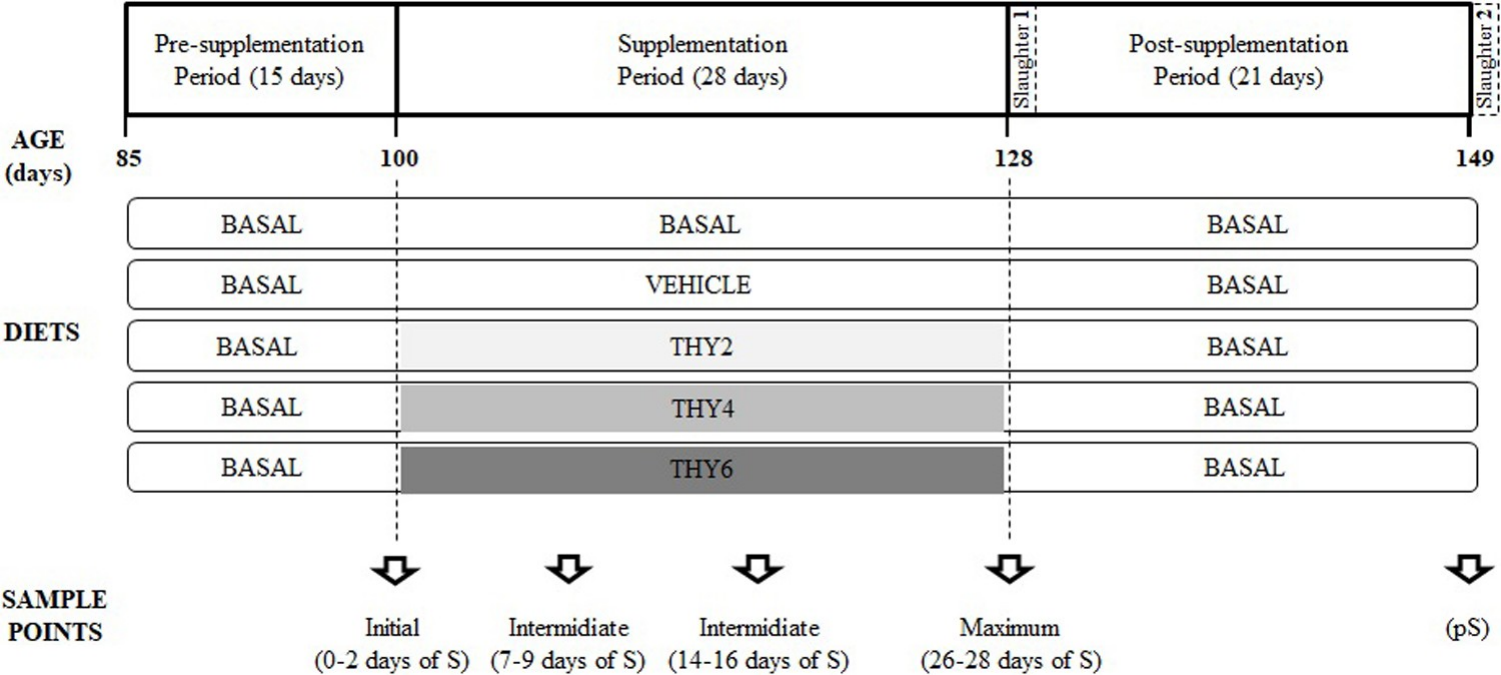
Timeline scheme. The age of the birds (days) is indicated on the horizontal axis. All birds received the basal diet between 85 and 100 days of age. From 100 to 128 days of age, females were subjected to chronic dietary supplementation (S). Five diets (N = 50; n = 10) were administrated (indicated with boxes): two controls (BASAL and VEHICLE) and three increasing doses of THY (THY2, THY4 and THY6, corresponding to 2, 4 and 6.25 g of THY/kg feed, respectively). Vertical dotted lines indicate changes in the diet of each experimental group. Once finished the supplementation period, half of the quail were slaughtered for histological analysis (Slaughter 1). The other half of the quail was subjected to a post-supplementation period of 21 days (pS) during which the basal diet was reestablished. Finished de pS period, the remaining birds were slaughtered (Slaughter 2). To assess dynamics five sample points were defined: one initial sample point; three sample points along the S period and one at the end of the pS period.

A total of 50 eighty-five days-old female quail of homogeneous body weights (251 ± 1 g; Mean ± SE) and in their egg-laying peak were selected from an initial group of 96 female individuals. All birds continued receiving a basal diet between 85 and 100 days of age and all variables (see below for details) were registered along this period (Pre-supplementation period, Fig 1). From 100 to 128 days of age (S period, Fig 1), females were randomly assigned to either a chronic dietary supplementation with one of 3 THY doses (THY2, THY4 and THY6; 10 females each) or to one of 2 control groups (basal diet, BASAL, and basal diet with vehicle solution, VEHICLE; 10 females each). Once finished the S period, half of the quail were slaughtered for histological analysis (n = 25; Slaughter 1, Fig 1) and the other half were subjected to a pS period of 21 days, during which all females received the BASAL diet (pS, Fig 1). After this period, the remaining 25 animals were also slaughtered for histological analysis (Slaughter 2, Fig 1). In this way, we assessed whether THY induced changes in variables indicative of tissue damage and if so, whether the potential damages could reverse after 3 weeks of supplement withdrawal.

To assess changes in egg yolk total FA composition, egg physical characteristics, laying rate, body weight, feed intake and general welfare, during the S and pS periods, five sampling points were defined (Sample points, Fig 1): one initial sample point (at 0 days of S); three sample points during the S period (at 7, 14 and 28 days of S); and one sample point at the end of the pS period (21 days of pS). It should be noted that for determinations of THY concentration, eggs and quail droppings were collected offset from the other variables measured by 2 days, thus were collected on days 2, 9, 16, and 26 days of S, and 17 days of pS, respectively.

Thus, a bi-factorial design combining the effects of the diet supplied (five levels) and time of sampling (five levels) was established for the variables studied.

### Dietary supplementation

Thymol was commercially obtained from Sigma-Aldrich (SAFC®, ⩾99%; FCC, Saint Louis, MO, USA). Supplemented quail were provided 2, 4 or 6.25 g of THY per kg of feed. For this end, THY was prepared in a 2.4, 4.8 and 7.5% w/v ethanolic solution and uniformly sprayed on fresh feed [16]. The feed was prepared and administrated daily, to minimize THY volatilization from feed before its consumption by females. The doses were selected considering two factors. The first, the concentration range for which the transfer of THY to the chicken and/or quail egg has been established [37,38]. Second, the concentrations of THY supplementation that have shown biological effects. For example, it was shown that THY2 reduces female quail fear responses when birds are exposed to stressful situations without affecting the bird´s locomotor activity [41]; and that THY6 enhances PUFA deposition to the detriment of SFA in yolk total fatty acids and triglycerides of the quail fertile egg [16]. In this context, THY4 is and intermediate dose between the last two. Nutrient and FA compositions of the diets supplied were identical between each other and are reported in S1, S2 and S3 Tables, and are in accordance with the National Research Council recommendation [42].

### Thymol quantification in egg yolk, quail droppings and feed

Egg yolks and droppings of each female were obtained at each sample point (Sample points, Fig 1) and THY concentration was immediately measured by head space-solid phase microextraction followed by gas chromatography-mass spectrometry (HS-SPME/GC-MS) according to Fernandez et al. [37]. SPME was performed with a manual holder with 100 um polydimethylsiloxane (PDMS) fiber. An aliquot of egg yolk (3 g), droppings (5 g) or feed (5 g) was put in a sealed 20 mL glass vial, spiked at the corresponding level with the standard m-cresol (Sigma-Aldrich; ≥ 99% (GC); Saint Louis, MO, USA) stock solution, and vortexed for 5 min. The vials were placed in a water bath and the PDMS fiber was exposed to the headspace for 30 min at 60°C and 5 min at 40°C for egg yolk and droppings or feed, respectively. The fiber was then inserted directly into the GC injector for desorption at 250°C for 10 min in splitless mode. Chromatography analysis was carried out in a Perkin Elmer Clarus 600 equipped with a PSSI injector and a quadrupole MS detector (Perkin Elmer, USA). Turbo Mass 5.4.2 software was used to control and acquire data from GC–MS. All the separations were performed using a Perkin Elmer fused silica DB 5 MS capillary column (60 m, 0.25 mm ID, 0.25 um film thickness), with High-purity helium (99.998%) as a carrier gas (49.6 psi). The splitless injection mode was selected. Electron-impact Ionization was carried out in the mass spectrometer under vacuum with 70-eV ionization energy. Samples were analyzed under the following chromatographic and MS detection conditions: initial oven temperature was set at 100°C (held for 2 min), and then raised to 230 at 10°C/min rate. A column head pressure of 14.99 psi and an injector temperature of 280°C were set. The GC transfer line was maintained at 250°C. The fiber was desorbed in the GC injector port for 10 min. Chromatograms were acquired in scan mode, which scans the quadrupole from m/z = 50 to m/z = 300 (scan time: 0.20 s, inter-scan time: 0.10 s). All quantitative analyses were performed in TIC mode. The compounds were identified by comparing their mass spectra with those of the libraries of the NIST MS search 2.0. The main components were further identified by co-injection of commercial standards.

### Total fatty acid analysis in egg yolks

Eggs were obtained at each sample point (Sample points, Fig 1) and stored at -20°C until yolk total fatty acids analysis. Lipids were extracted from the yolk following homogenization in a suitable excess of chloroform/methanol (2:1 v/v) [43]. The solvents were removed under reduced pressure in a rotary evaporator. Lipids were subjected to alkaline saponification (1 mol/L potassium hydroxide in methanol), and the unsaponifiable matter was extracted with *n*-hexane. The fatty acid methyl esters (FAMEs) were prepared by transmethylation through treatment with 1 mol/L sulfuric acid in methanol and analyzed by gas chromatography/mass spectrometry (GC/MS) [16,44]. All chemicals used in this study were reagent-grade commercial products. FAMEs were analyzed by gas chromatography on a 60 m fused capillary column with an internal diameter of 0.25 mm (PerkinElmer Elite-WAX Polyethylene Glycol). The analysis was performed on a PerkinElmer Clarus® 600 GC/MS system equipped with a flame ionization detector (Waltham, MA, USA). Helium was used as carrier gas (constant flow of 49.6 psi). The injection port temperature was 250°C and the detector temperature was 250°C. The oven temperature was initially held at 180°C for 5 min, then increased at 4°C/min to 200°C and held for 5 min and finally increased at 3°C/min to 230°C and held for 25 min. Peak identification was carried out by comparing the known retention times for the fatty acids reported with the temperature program and the chromatographic system used. A solution of known concentration of nonadecanoic acid methyl ester (Sigma Aldrich, ≥98.0% (GC); Saint Louis, MO, USA) as was used internal standard to estimate the content of each fatty acid in the sample.

### Female performance traits

Female performance was evaluated through the measurement of body weight, feed intake, egg laying rate, egg physical characteristics and a general welfare quality assessment. Body weight was measured once a week throughout the experimental period. Daily feed intake (DFI) was estimated as the difference between the amount of feed supplied to each animal (60 g) and the rest that remained in the feeders the next day (g of feed/day/quail) [45,46]. Egg laying was registered daily and the weekly laying rate was calculated as: (number of eggs / 7 days) x 100. Egg weight, quality characteristics (such as intact, membranous, soft shell and broken shell), morphometric characteristics (egg shape index = (egg width / egg length) x 100) and percentage of constituents (egg yolk, albumen and shell) were registered at each sampling point [15,47].

A welfare assessment based on observations of physical characteristics was made. Female skin lesions and plumage status were evaluated following a procedure proposed by Pellegrini et al. [48] that is an adapted version of the protocol developed by the Welfare Quality consortium [49]. Briefly, skin lesions, which include wounds that have not healed in the legs, rear end, chest, cloacae and wings were determined using a score scale from 0 to 2, where "0" represents no lesions or scratches, "1" represents at least one lesion < 0.5 cm diameter or less than 3 pecks (punctiform damage ~ 0.1 cm of diameter) or scratches, and "2" reflects one lesion ≥ 0.5 cm of diameter or more than 3 pecks or scratches. Plumage damage was also determined using a score scale from 0 to 2 as follows: "0" represents individuals with no plumage damage or slight wear (only single feathers lacking), "1" represent individuals with one or more body parts that have moderate wear (i.e. damaged feathers worn or deformed) or one or more featherless areas < 1.5 cm in diameter at the larger extent and "2" corresponded to individuals that have at least one featherless area > 1.5 cm in diameter at the largest extent. Foot pad dermatitis, for which both feet were analyzed and the foot with the worst condition was scored according to the following: "0" representing feet intact, no or minimal proliferation of epithelium, "1" corresponded to necrosis or proliferation of epithelium or chronic bumble foot with no or moderate swelling, and "2" indicated swollen dorsally visible. Eye pathologies, which include swelling of the eyelids and the skin around the eyes, closure of the eye/eyes and discharge from the eyes were classified as "0" when no evidence of eye pathologies were observed or "1" if a there were eye pathologies. Additionally, dirt from the legs, cloacae and belly was examined for signs of diarrhea potentially caused by dietary supplementation.

### Liver histological analysis

At each slaughter point birds were euthanized by decapitation and livers were removed and stored in buffered formalin at 10%, processed routinely and stained with hematoxylin and eosin (H&E) and periodic acid-Schiff (PAS). Each slide was examined blinded, with a light microscope (Olympus X-785) and photographed with a digital camera (Moticam Camera 2300, 3 Megapixels). To evaluate histological alterations of liver, tissue was divided into 8 random equal areas to ensure there was no overlapping of the studied areas. For each area, the extension and number of each alteration were recorded with a microscope at 40X magnitude. Histopathological index of liver (HI_liv_) were estimated using a semi-quantitative protocol following [50], modified by our colleagues [51]. Briefly, alterations were classified into four major reaction patterns each one of those including alterations that concern to different functional units of the liver or to the whole organ: RP1, circulatory disturbances (dilatation of sinusoids, vascular congestion, hemorrhage); RP2, regressive changes (steatosis or fatty degeneration, hydropic degeneration, nuclear alteration, fibrosis, necrosis); RP3, progressive changes (oval cells, cell hypertrophy and hyperplasia); and RP4, liver inflammation (leukocyte infiltration). Then, for each RP, an index was calculated based on two factors: the pathological importance of the lesions (importance factor, W) (range 1–3) and the extension of pathological change (score value, a) from 0 (unchanged) to 8 (extreme occurrence). Finally, a total liver histopathological index (HI_liv_) was calculated by adding the single RP liver indices of each individual quail. A greater value of (HI_liv_) reflects the most severely affected individual.

### Statistical analysis

General and generalized linear mixed models were used in order to analyze the effects of the diet supplied (5 levels of factor) and time of sampling (5 levels of factor) throughout experimental manipulation. The diet supplied and time of sampling were included as fixed effects and female identity was included as a random effect for THY concentration, FA composition, feed intake, body weight, morphometric variables and percentage of egg constituents. For histological analysis, a two-way ANOVA including the diet supplied and the slaughter batch (28 days of S or 21 days of pS) as effects was used. Egg-laying was analyzed as cumulative number of eggs using a one-way ANOVA with the diet supplied as unique factor. Data were analyzed according to normal distribution unless stated otherwise and assumptions of the tests were verified. The variables THY concentration, weekly body weight, SFA, arachidonic, eicosapentaenoic and docosapentaenoic acids of egg yolk were transformed before analysis (log10 was used for THY concentration in the yolk and weekly body weight, root square for THY concentration in the droppings, and Logit for all other variables). Palmitic and stearic acids and egg weight that were analyzed according to gamma distribution. A P-value of <0.05 was considered to represent significant differences. DGC test was used for post hoc comparisons. All statistical analyses were performed with ‘R’ (The R Foundation for Statistical Computing) through a user-friendly interface implemented in InfoStat [52].

## Results

### Thymol quantification in egg yolks and droppings

**Fig 2:**
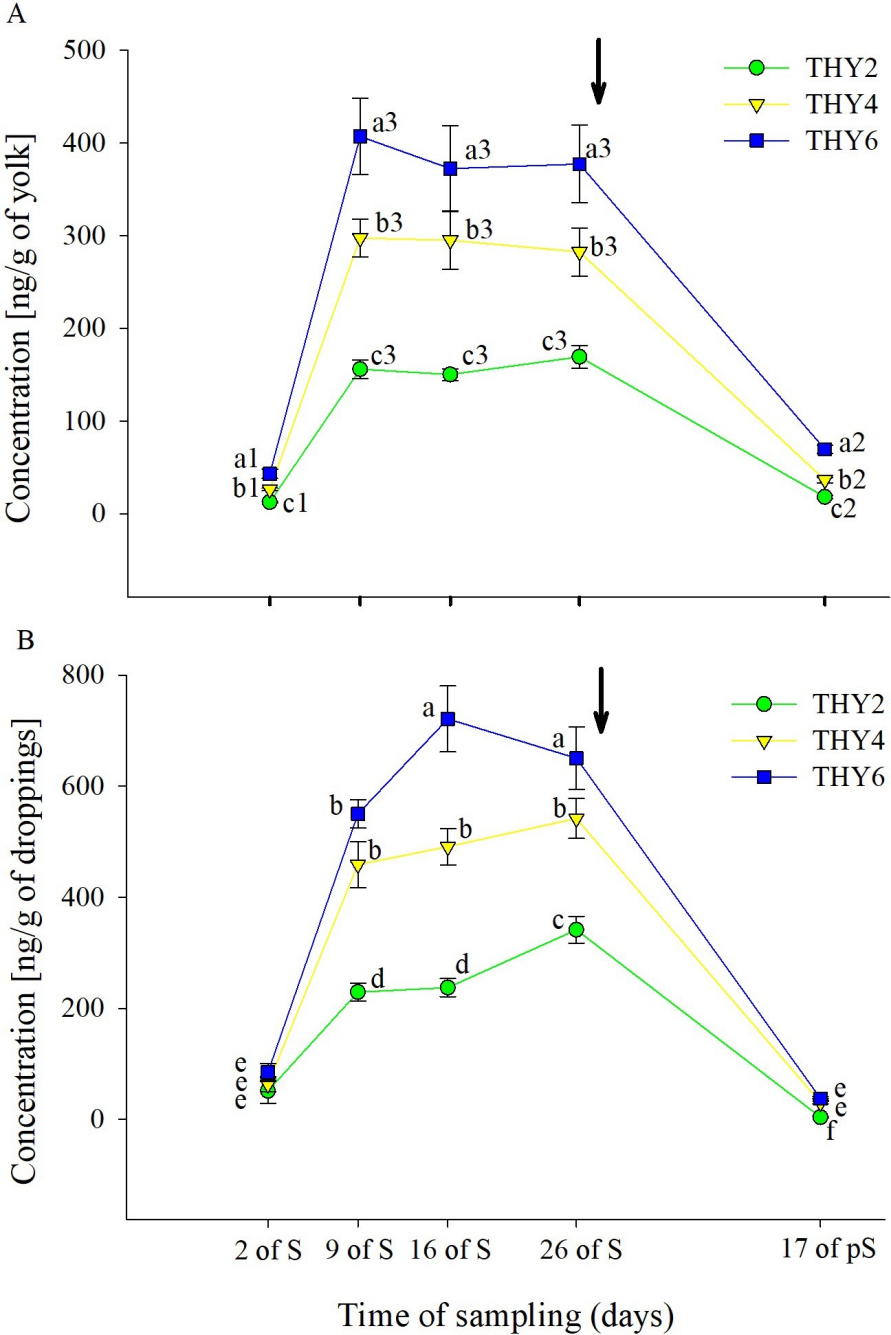
Thymol concentration in quail egg yolk and droppings. Mean ± SEM of thymol concentration (ng/g) in the (A) egg yolk and (B) droppings from females fed on diets with 2, 4, and 6.25 g of thymol/kg of feed (THY2, THY4 and THY6, respectively) at 2, 9, 16, and 26 days of the supplementation period (S) and after 17 days of supplement withdrawal (pS). Arrow pointing down indicates the end of the S period. In Fig 2A different letters (a, b, c) and numbers (1, 2, 3) indicate statistical differences for the diet supplied and time of sampling, respectively. In Fig 2B different letters (a, b, c, d, e, f) indicate statistical differences for the interaction. The same 15 females (5 per THY supplemented diet) were sampled on each of the five sample points (75 eggs and 45 droppings samples total). BASAL and VEHICLE samples were evaluated at the beginning and end of the day at each sample point to assure the absence of THY.

The dose-dependent effects of the diet supplied over the time of sampling on THY concentration in the egg yolk and droppings are shown in Fig 2. In the egg yolk, significant effects (Table 1 and Fig 2A) of both the diet supplied and time of sampling on THY concentration were found. Post-hoc analysis showed that THY concentration increased on 9 days of S. Subsequently, the concentration reached a plateau that lasted until the end of the S period. After supplement withdrawal, THY concentration decreased. Regarding the THY doses tested, it was found that the higher the dose supplemented, the higher the concentration it is detected. In dropping samples, a significant interaction (Table 1 and Fig 2B) between the diet supplied and time of sampling was observed, with the same general pattern described in the egg yolk with the exception that THY6 was higher than THY4 only at 16 and 26 days of S and that THY2 was higher at 26 days than at 16 days of S.

**Table 1:** Statistical information corresponding to the effects of the diet supplied, time of sampling and their interaction on thymol (THY) concentration in quail egg yolk and droppings and concentration of egg yolk total fatty acids.

Variables	N; n		Analysis of variance factors and degrees of freedom		
			Diet supplied (D)	Time of sampling (T)	D x T
THY concentration in the egg yolk	75;5	F	160.86 (dF = 2;12)	505.77 (dF = 4; 48)	1.91 (dF = 8; 48)
		P	0.0001	0.0001	0.0805
THY concentration in the droppings	45;3	F	84.99 (dF = 2; 6)	314.08 (dF = 4; 24)	5.12 (dF = 8; 24)
		P	0.0001	0.0001	0.0008
Saturated Fatty Acids (SFA)	125; 5	F	20.22 (dF = 4;20)	24.07 (dF = 4; 80)	7.57 (dF = 16; 80)
		P	0.0001	0.0001	0.0001
Monounsaturated Fatty Acids (MUFA)	125; 5	F	0.61 (dF = 4;20)	1.03 (dF = 4; 80)	0.45 (dF = 16; 80)
		P	0.67	0.4	0.96
Polyunsaturated Fatty Acids (PUFA)	125; 5	F	18.27 (dF = 4; 20)	10.27 (dF = 4; 80)	4.40 (dF = 16; 80)
		P	0.0001	0.0001	0.0001
Palmitic acid (16:0)	125; 5	χ^2^	15.90 (dF = 4; 20)	34.19 (dF = 4; 80)	89.58 (dF = 16; 80)
		P	0.003	0.0001	0.0001
Stearic acid (18:0)	125; 5	χ^2^	16.14 (dF = 4; 20)	27.51 (dF = 4; 80)	45.55 (dF = 16; 80)
		P	0.003	0.0001	0.0001
Palmitoleic acid (16:1)	125; 5	F	3.23 (dF = 4; 20)	1.46 (dF = 4; 80)	3.28 (dF = 16; 80)
		P	0.03	0.22	0.0002
Oleic acid (18:1)	125; 5	F	1.08 (dF = 4; 20)	0.73 (dF = 4; 80)	0.47 (dF = 16; 80)
		P	0.4	0.58	0.96
Linoleic acid (18:2)	125; 5	F	0.90 (dF = 4; 20)	0.61 (dF = 4; 80)	1.03 (dF = 16; 80)
		P	0.48	0.66	0.44
Linolenic acid (18:3)	125; 5	F	1657.30 (dF = 4; 20)	716.39 (dF = 4; 80)	306.90 (dF = 16; 80)
		P	0.0001	0.0001	0.0001
Arachidonic acid (20:4)	125; 5	F	33.86 (dF = 4; 20)	8.93 (dF = 4; 80)	5.53 (dF = 16; 80)
		P	0.0001	0.0001	0.0001
Docosahexaenoic acid (22:6)	125; 5	F	92.43 (dF = 4; 20)	49.46 (dF = 4; 80)	14.09 (dF = 16; 80)
		P	0.0001	0.0001	0.0001
Eicosapentaenoic acid (20:5)	125; 5	F	9.16 (dF = 4; 20)	12.34 (dF = 4; 80)	3.67 (dF = 16; 80)
		P	0.0002	0.0001	0.0001
Docosapentaenoic acid (22:5)	125; 5	F	26.25 (dF = 4; 20)	30.06 (dF = 4; 80)	3.40 (dF = 16; 80)
		P	0.0001	0.0001	0.0002

### Changes in egg yolk total fatty acid composition

**Fig 3:**
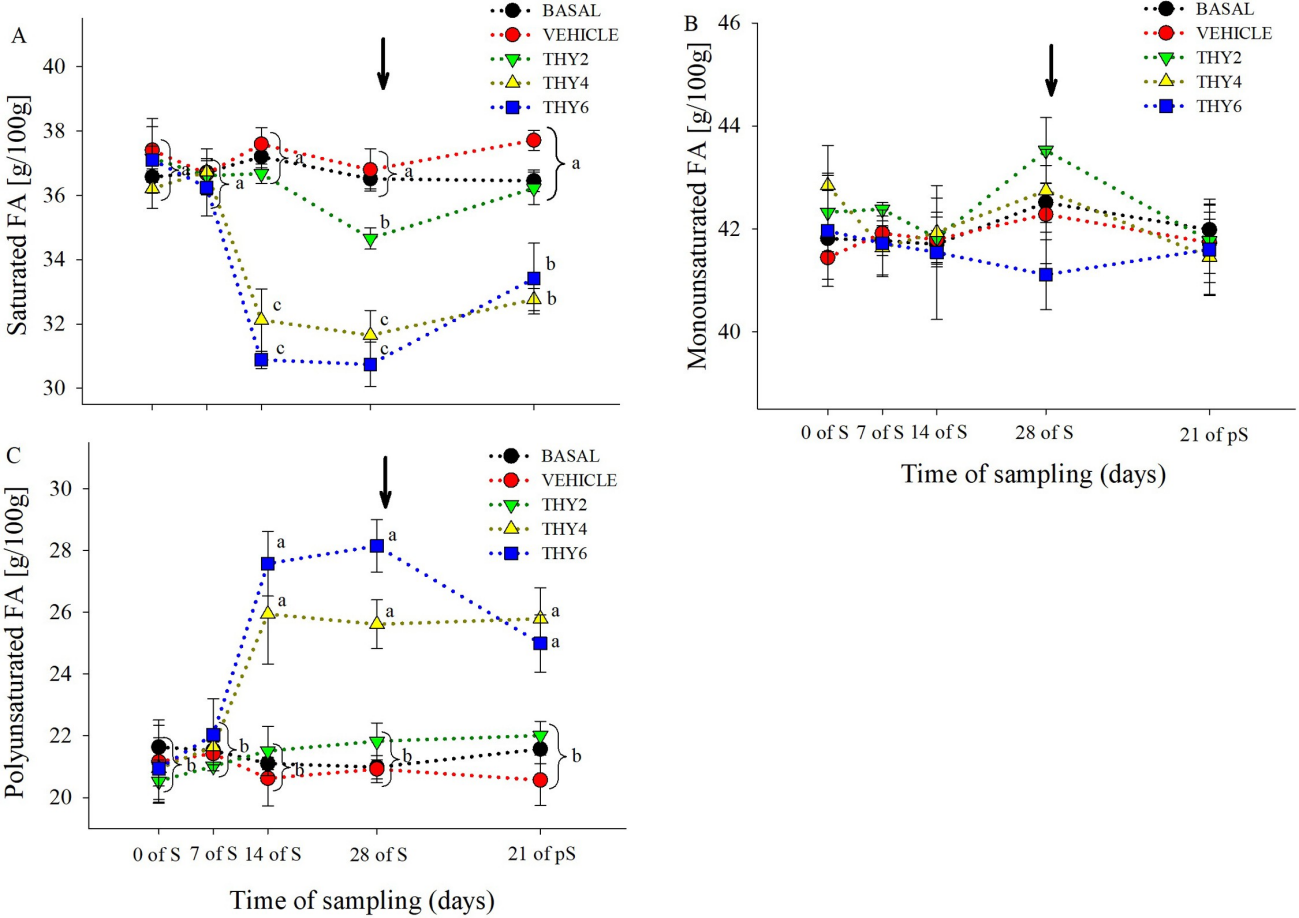
Concentration of saturated, monounsaturated and polyunsaturated fatty acids of quail egg yolk. Mean ± SEM of content (g/100 g FAME) of (A) saturated (myristic + pentadecanoic + palmitic + stearic acids), (B) monounsaturated (palmitoleic + oleic acids) and (C) polyunsaturated (linoleic + linolenic + arachidonic + docosahexaenoic + eicosapentaenoic + docosapentaenoic acids) fatty acids of quail egg yolk laid by females fed on the control diets (BASAL and VEHICLE) or supplemented with 2, 4, and 6.25 g of thymol/kg of feed (THY2, THY4 and THY6, respectively) at 0, 7, 14, and 28 days of the supplementation period (S) and after 21 days of supplement withdrawal (pS). Arrow pointing down indicates the end of the S period. Brackets refer to measurements within a time of sampling. Different letters (a, b, c, d) indicate statistical differences. The same 25 females (5 per diet supplied) were analyzed on each of the five sample points (125 eggs total).

**Fig 4:**
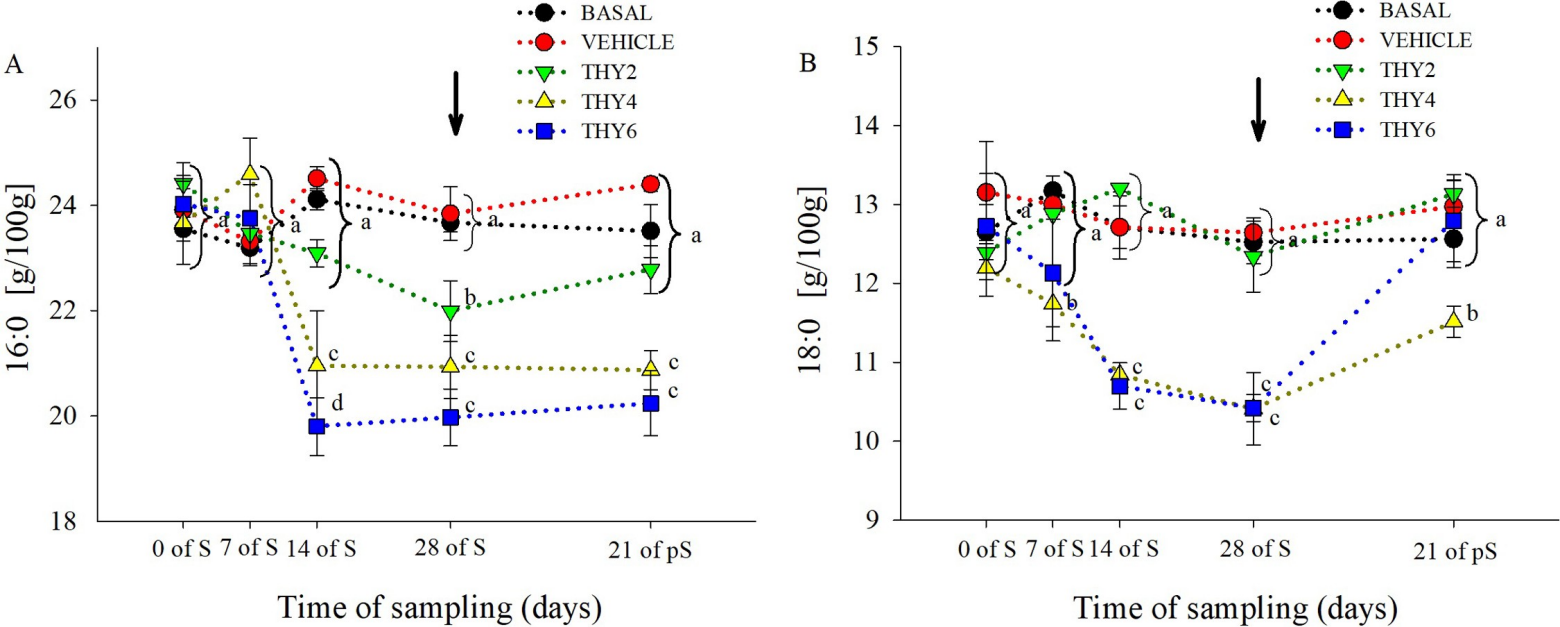
Concentration of saturated fatty acids of quail egg yolk. Mean ± SEM of content (g/100 g FAME) of (A) palmitic (16:0) and (B) stearic acids (18:0) of quail egg yolk laid by females fed on the control diets (BASAL and VEHICLE) or supplemented with 2, 4, and 6.25 g of thymol/kg of feed (THY2, THY4 and THY6, respectively) at 0, 7, 14, and 28 days of the supplementation period (S) and after 21 days of supplement withdrawal (pS). Arrow pointing down indicates the end of the S period. Different letters (a, b, c) indicate statistical differences. Brackets refer to measurements within a time of sampling. The same 25 females (5 per diet supplied) were analyzed on each of the five sample points (125 eggs total).

**Fig 5:**
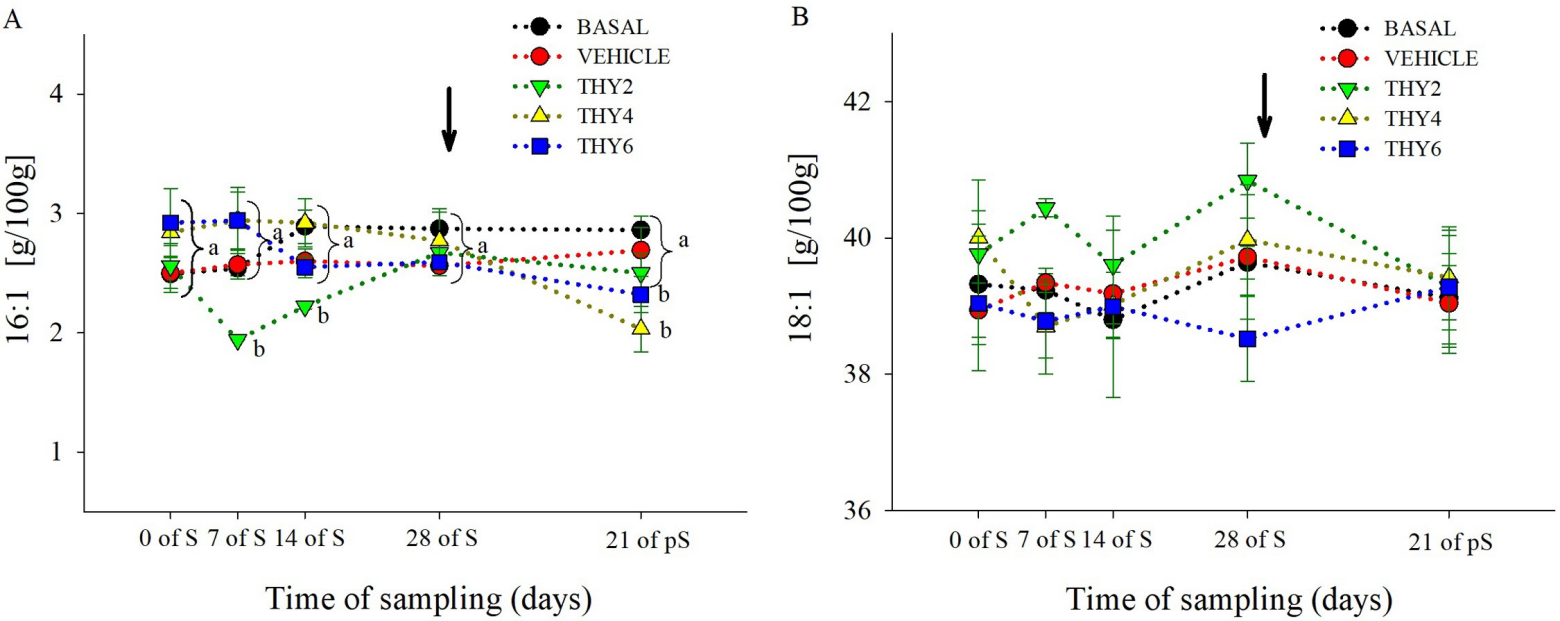
Concentration of monounsaturated fatty acids of quail egg yolk. Mean ± SEM of content (g/100 g FAME) of (A) palmitoleic (16:1) and (B) oleic acids (18:1) of quail egg yolk laid by females fed on the control diets (BASAL and VEHICLE) or supplemented with 2, 4, and 6.25 g of thymol/kg of feed (THY2, THY4 and THY6, respectively) at 0, 7, 14, and 28 days of the supplementation period (S) and after 21 days of supplement withdrawal (pS). Arrow pointing down indicates the end of the S period. Different letters (a, b) indicate statistical differences. Brackets refer to measurements within a time of sampling. The same 25 females (5 per diet supplied) were analyzed on each of the five sample points (125 eggs total).

**Fig 6:**
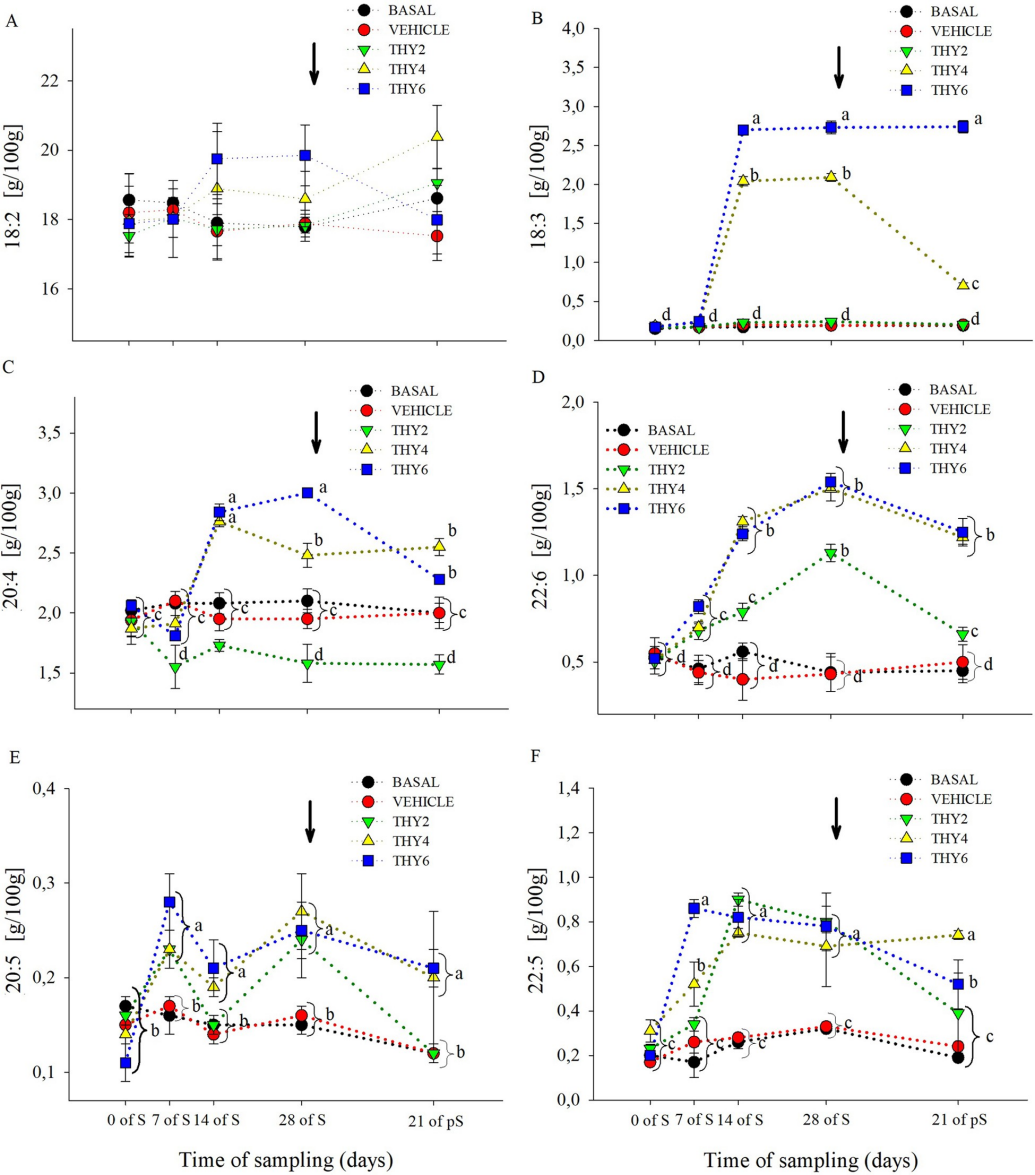
Concentration polyunsaturated fatty acids of quail egg yolk. Mean ± SEM of content (g/100 g FAME) of (A) linoleic (18:2), (B) linolenic (18:3), (C) arachidonic (20:4), (D) docosahexaenoic (22:6), (E) eicosapentaenoic (20:5) and (F) docosapentaenoic (22:5) acids of quail egg yolk laid by females fed on the control diets (BASAL and VEHICLE) or supplemented with 2, 4, and 6.25 g of thymol/kg of feed (THY2, THY4 and THY6, respectively) at 0, 7, 14, and 28 days of the supplementation period (S) and after 21 days of supplement withdrawal (pS). Arrow pointing down indicates the end of the S period. Different letters (a, b) indicate statistical differences. Brackets refer to measurements within a time of sampling. The same 25 females (5 per diet supplied) were analyzed on each of the five sample points (125 eggs total).

The dose-dependent effects of the diet supplied over the time of sampling on egg yolk FA concentration are shown in Figs 3–6. Significant effects (Table 1) of the diet supplied, time of sampling and their interaction was found for SFA and PUFA. As expected, post-hoc analysis showed that control (BASAL and VEHICLE) females maintained constant SFA and PUFA values throughout experimental manipulation (Fig 3A–3C). On the contrary, along the S period THY4 and THY6 induced PUFA increments that remained enhanced even at 21 days of pS, while THY2 failed to increase PUFA (Fig 3C). Specifically, all THY supplemented diets increased 22:6 and 20:5 from 7 days of S on, but only THY4 and THY6 showed higher concentrations of 18:3 and 20:4, from 14 days of S onwards (Fig 6B–6E). In addition, THY2 and THY4 required 14 days of S for increasing 22:5, while THY6 needed only 7 days of S for achieving similar changes (Fig 6F). On the other hand, THY2 required 28 days of S to reduce the SFA concentration, while THY4 and THY6 only needed 14 days of S for a reduction of similar and even greater magnitude, respectively. However, after 21 days of pS the aforementioned effect disappeared in THY2 but not in THY4 and THY6 (Fig 3A). Concomitantly, reduced 16:0 and 18:0 concentrations were achieved by 14 days of S in THY4 and THY6 and by 28 days of S in THY2. After 21 days of pS those effects continued in all diets, except for the 16:0 and 18:0 concentrations in THY2 and THY6, respectively, that returned to initial levels (Fig 4A and 4B). No differences were observed between the diet supplied, time of sampling or their interaction in MUFA (Fig 3B). However, a transient decrease of 16:1 was observed by 7 and 14 days of S in THY2 (Fig 5A and 5B) and at 21 days of pS in THY4 and THY6 respect to their diet and time counterparts.

### Female performance traits

The effects of the diet supplied over the time of sampling on variables related to performance (body weight, feed intake, egg laying, morphometric characteristics and constituents of eggs) are shown in Table 2. Because no effects of the diet supplied, time of sampling or interaction were found, the Table is only showing the overall averages. Considering that no differences in the daily feed intake was observed between treatments (30.3 ± 1 g/day/animal), it can be assumed that the amount of THY consumed was proportional to the dose supplemented. By measures of THY concentration in the feed (S4 and S5 Tables) we were able to estimate a daily THY intake of 0.26, 0.70 and 1.2 mg/day/animal for THY2, THY4 and THY6, respectively. Thus, concentrations of THY transferred to the egg yolk and droppings during the plateau respectively correspond to ~0.13–0.25% and ~1.57–3.47% of the amount THY ingested.

Regarding general welfare, 100% of the birds of all treatments showed optimal plumage on the back and rump and around the cloacae, no skin lesions were observed, and only minimal proliferation of epithelium were observed (category 0) throughout S and pS periods. In relation to plumage of the neck and head, eye pathologies and dirt in the cloacae and legs, only a few isolated cases of category 1 were observed and were not associated with any particular treatment.

**Table 2:** Overall mean values (± SEM) and statistical information regarding performance related variables from female quail fed with control diets or supplemented with thymol (Diet supplied) over five time points (Time of sampling).

Variables	Mean ± SEM			*p*-Value		
				Diet supplied (D)	Time of Sampling (T)	D x T
Weekly Body Weight per individual (g)	254	±	1	0.83	0.17	0.92
Weekly Feed Intake per individual (g)	212	±	2	0.55	0.11	0.88
Cumulative number of eggs	42	±	1	0.39	-	-
Egg weight (g)	11.3	±	0.1	0.50	0.49	0.26
Egg shell (%)	14.4	±	0.2	0.83	0.54	0.71
Egg yolk (%)	37.0	±	0.4	0.90	0.87	0.99
Egg albumen (%)	48.6	±	0.5	0.95	0.96	0.99
Egg shape index (%) *	79.5	±	0.3	0.73	0.46	0.77

Considering that no effects of the diet supplied, time of sampling or their interaction were found, only overall means are shown. Two control (BASAL and VEHICLE) and three supplemented diets (2, 4, and 6.25 g of thymol/kg of feed) were used. The five sampling time points used were at 0, 7, 14, and 28 days of the supplementation period and after 21 days of supplement withdrawal.

* Egg shape index = (egg width / egg length) x 100

### Liver histological analysis

**Fig 7:**
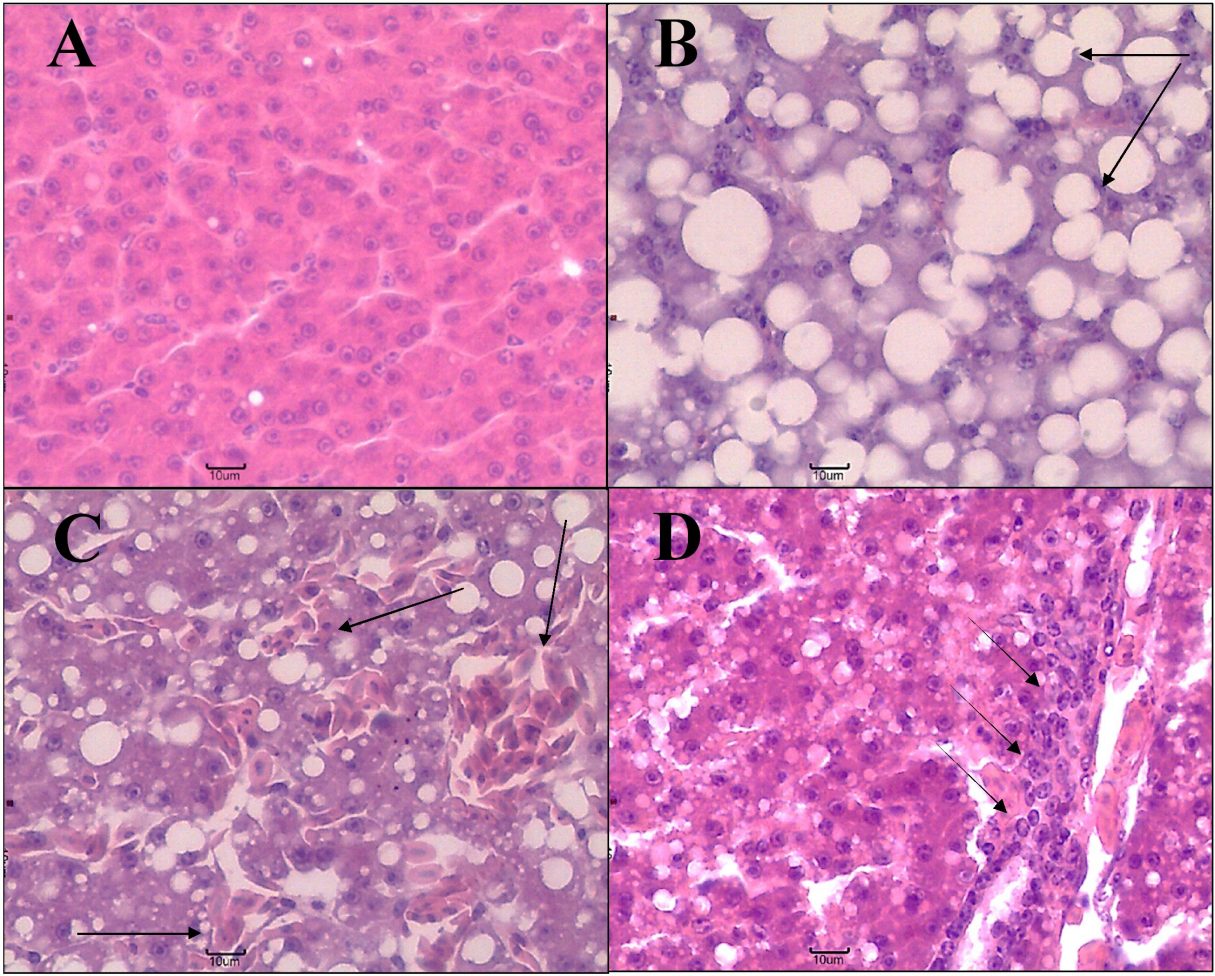
Example photomicrographs of quail liver 40X. (A) Normal liver, (B) liver with steatosis or fatty degeneration, (C) vascular congestion and dilatation of sinusoids, and (D) oval cell focus. Arrows point to representative examples of the corresponding alteration in the photograph.

**Fig 8:**
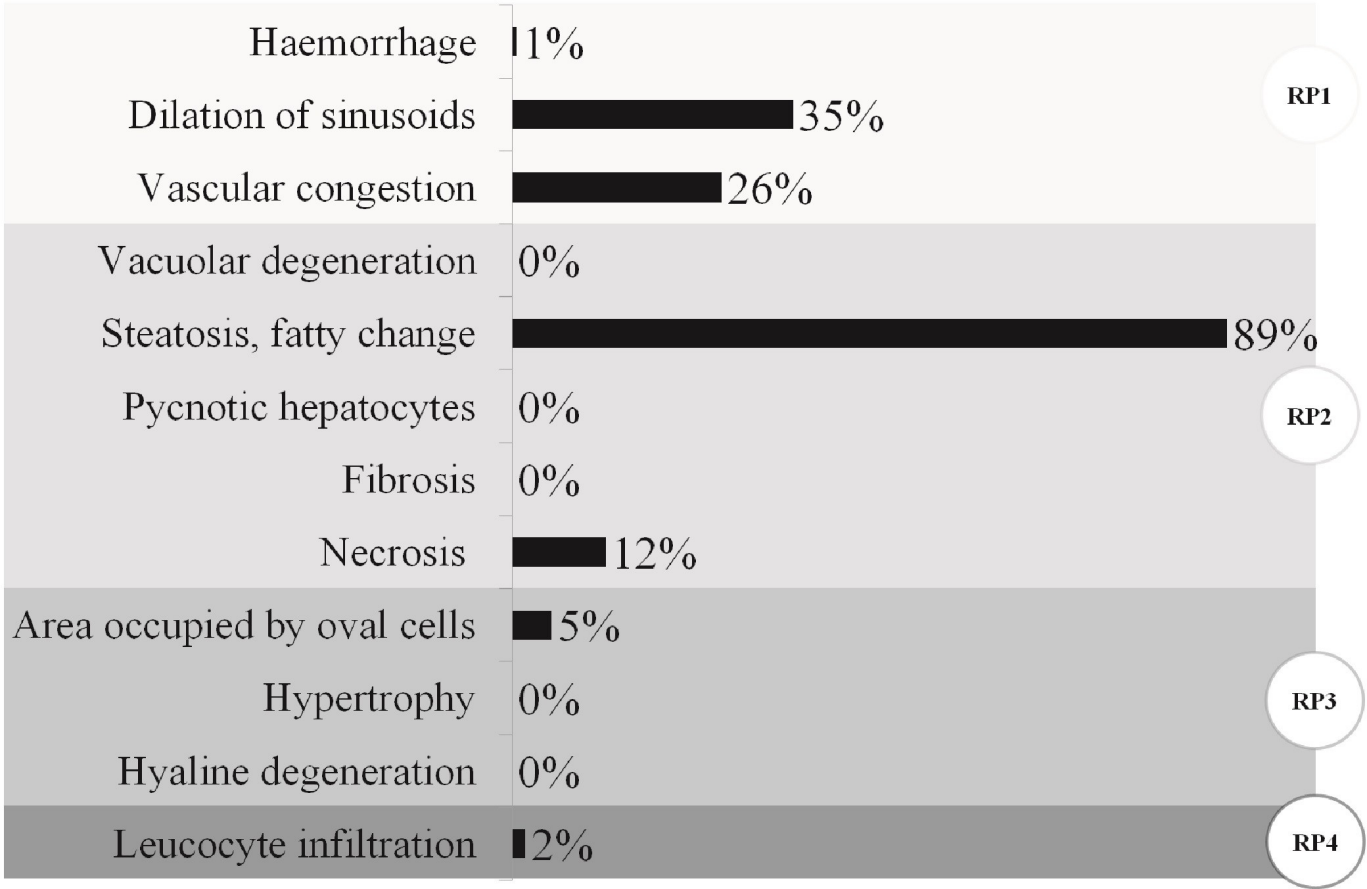
Frequency of histopathological alterations registered in all livers analyzed. The reaction pattern (RP) to which the alterations correspond is indicated with different intensity of gray and a circular label: RP1 (circulatory disturbances), RP2 (regressive changes), RP3 (progressive changes), RP4 (inflammation) (n = 5; N = 45, only 5 birds from the basal diet were studied).

As a general description of the state of experimental animals, representative photomicrographs and the frequency of histopathological alterations registered in all livers analyzed regardless of the diet supplied or sample point are presented in Figs 7 and 8, respectively. The presence of steatosis or fatty degeneration was recorded in 89% of the livers evaluated (Figs 7B and 8). Sinusoidal dilatation and vascular congestion (both considered reversible changes) were observed respectively in 35% and 26% of the samples (Figs 7C and 8). A low number of small necrotic foci (12%) and foci of oval cells (associated with tissue regeneration) were found (Figs 7D and 8). Both the leukocyte infiltration and the pyknotic nuclei were recorded at low frequency (<5%). Thus, among histopathological alterations, regressive changes (PR2: steatosis or fatty degeneration and necrosis) registered the highest frequency, followed by circulatory alterations (PR1: sinusoidal dilatation and vascular congestion) and finally progressive and inflammatory changes (PR3: foci of oval cells and PR4: inflammation) (Fig 8).

The effects of dietary supplementation on liver histopathological indices of animals that had gone through 28 days of S and the pS period are shown in Table 3. Because no treatment effects were found, only overall averages are shown. Moreover, as histopathological alterations corresponding to Rp3 and Rp4 were observed only in a few individuals that were not associated with any particular treatment, *p*-values in those cases were not calculated. Total histopathological indices varied between 17.6 and 27.6.

**Table 3:** Overall mean values (± SEM) and statistical information regarding liver histopathological indices by reaction pattern and total liver index for female quail fed with control diets or supplemented with thymol (Diet supplied) slaughtered at two time points (Slaughter batch).

Index	Mean ± SEM			*p*-Value		
				Diet supplied (D)	Slaughter batch (B)	D x B
HI_Liv.Rp1_	4,71	±	0,41	0.37	0.74	0.67
HI_Liv.Rp2_	16,80	±	0,65	0.19	0.57	0.81
HI_Liv.Rp3_	0,80	±	0,24	-	-	-
HI_Liv.Rp4_	0,27	±	0,27	-	-	-
HI_Liv_	22,58	±	0,90	0.15	0.37	0.78

Considering that no effects of the diet supplied, slaughter batch or their interaction were found, only overall means are shown. Two control (BASAL and VEHICLE) and three supplemented diets (2, 4, and 6.25 g of thymol/kg of feed). Two slaughter batches were assessed, the first, once finished the supplementation period, and a second after the post-supplementation period of 21 days. HI = Histopathological index. Liv = Liver. RP = Reaction Pattern; RP1 (circulatory disturbances), RP2 (regressive changes), RP3 (progressive changes), RP4 (inflammation). The reaction pattern to which the indices correspond is indicated with different grey intensity (n = 5; N = 45, only 5 birds from the basal diet were studied).

## Discussion

The main contribution of the present work is to show that both the dose of THY and the duration of the S period directly modulate the FA concentrations in quail eggs showing positive changes that remain apparent even after 21 days of THY withdrawal. Interestingly, the phenomenon was observed without changes in liver histology or impairments in any of the female productive traits registered. Furthermore, the bioavailability of THY both in the egg yolk and in droppings (i.e. an indicator of changes in circulating levels of THY) was also directly related to the concentration of THY and the duration of the supplementation.

In general, THY supplementation induced increments in PUFA concentration with a decrement in SFA in the egg yolk that were evident after 14 days of supplementation. These changes in the FA profile are consistent with an improved nutritional quality of eggs both from human nutrition standpoint (i.e. for consumption of healthy eggs; [17,18]) and for early avian nutrition and hatching success [15,16,20].

By simultaneously assessing the dynamics of changes in THY and FA concentration along S and pS periods in several doses of THY, we were able to display the relations between the pattern of change in each variable (time-dose response curves) that may account for the multiple possible roles of THY in vivo. In the initial phase of the S period, both THY and PUFA concentrations increased over time. Afterwards, in a second phase, although THY concentration plateaued both in the egg yolk and droppings, PUFA concentration in the yolk continued increasing. Likewise, after THY supplementation withdrawal (pS period), while THY concentration was already depleted, PUFA concentration remain increased. An inverse relation of patterns was observed for SFA. Thus, these results provide evidence towards the hypothesis that THY modulation of FA profile is beyond its direct antioxidant activity (i.e. scavenging free radicals or chelating metal ions; [53–56]). The further FA-modulation evidenced in the second phase and during pS can be understood under different rational. First, FA-modulation can be considered a result of the sum of effects of the direct and indirect THY antioxidant activity [8,13,57–59] in different organs [9,60–62]. Second, THY properties could affect other metabolic pathways that could conduce to PUFA increments [13,63–66]. Third, FA-modulation mediated by the PUFA [67,68], specifically, in the pS period. In the following paragraphs these three alternatives are explained in detailed.

In regard to the potential direct antioxidant effect of THY in target tissues (i.e. yolk) it is important to take into consideration the minimum concentration necessary to provide meaningful effects. In our study the highest concentrations of THY detected was in the range of 406 ng/g of egg yolk similar to other studies [34,38], which is equivalent to ~3.05 uM. It has been stated that given a total antioxidant value in human plasma of over 103 mM, a minimum concentration of 20–50 mM (~36%) of an additional antioxidant from dietary sources would be required to make a significant contribution to systemic antioxidant capacity [69]. A similar situation would be found in chickens where antioxidant concentrations in plasma such as vitamin C and vitamin E would be in a range of 61–66 uM and 13–15 uM, respectively, and total antioxidant value in plasma could vary from 336.9 mM up to 740–830 mM, considering that dietary polyphenols result in unconjugated serum levels of up to 1 mM [70]. In the same way, it has been demonstrated that a concentration of 278 ug of THY/g of yolk was needed to be added to a control yolk to display an antioxidant activity equivalent to that of a yolk from a thyme treated hen [14]. Although these authors attributed the increased antioxidant status and FA modulation observed in vivo to the activity of the other components in thyme, their result also could be associated with THY involvement in other mechanisms than the direct antioxidant activity in the body. It is important to mention that these authors were not able to analyze the actual concentration of THY in eggs since at that moment they lacked a method to do so. It has been proposed that if polyphenols have direct antioxidant effects *in vivo*, they might be capable of exerting such effects more likely within the gastrointestinal tract, where high concentration of polyphenols may come into direct contact with cells without having undergone absorption and metabolism [60]. In this context, dietary grape seed proanthocyanidins in rats [61] and piglets [62] has been shown to suppress oxidative stress in the intestinal mucosa, improving general health status. This hypothesis would also be in accordance with the contention that the chain reaction included in the oxidation of consumed lipids could be inhibited by the transfer of natural antioxidants such as THY into the female body by feeding, consequently decreasing the oxidation of constituents transferred into the egg yolk and helping to maintain PUFA [71]. Thus, THY direct antioxidant activity at gastrointestinal tract level would be plausible, helping to promote the FA-modulation observed herein. However, the relatively low concentration of THY detected in target tissues seems to shift the rational from a focus on direct antioxidant properties to a focus on indirect antioxidant properties, biotransformation, signaling transduction and gene expression regulation.

The indirect antioxidant effects of THY are based on the potential pro-oxidant activity of this phenol. In several *in vitro* experimental systems THY has demonstrated to enhance reactive oxygen species (ROS) formation, in a dose and time dependent manner [72–74]. In this regard, recent studies have shown that administration of moderate doses of epigallocatechin gallate (EGCG) to mice can produce ROS, which activates the nuclear factor Nrf-2-mediated induction of antioxidant and other cytoprotective enzymes [75–77]. Analogous results have been reported in quail supplemented with EGCG [78]. Whether THY can exert a similar mechanism in vivo must be proved, but it would be consistent with its known effects on antioxidant enzymes activity in tissues with high PUFA absolute content (serum, liver, thigh muscle, brain). For example, in broiler [13] and rats [79], it has been demonstrated that dietary THY boosted glutathione peroxidase and superoxide dismutase activities along with a decrease in malondialdehyde levels and an increase in PUFA concentrations, as observed herein. Similar results have been found in chickens that were fed *Cinnamomum cassia* [80]. Thus, depending on the scale, pro-oxidant effects can be beneficial and induce FA profile changes.

Other metabolic pathways could also be affected by THY supplementation such as lipids digestion and fatty acids bioconversion. As it is widely known, birds, like other animals, can only acquire PUFA precursors (linoleic and alpha-linolenic acids) through the diet, since they are unable to synthesize them *de novo* [81]. Once these precursors are in the animal body, their long chain derivatives (LCPUFA) can be synthesized. Since the supplemented diets were equal in composition (S3 Table), it could be conceived that THY promotes greater bioavailability of PUFA precursors through increasing digestive enzyme activities and feed efficiency, which has been shown to be a dose-dependent phenomena [2,13,82]. In addition, THY could promote greater bioconversion of these precursors in their LCPUFA through increasing delta-desaturases enzymes activity in the liver. On this subject, it has been demonstrated that hydroxytyrosol (a natural polyphenol) supplementation in mice enhances liver delta-desaturases, associated not only with an improvement in n-3 PUFA concentrations but also with a decrease in oxidative stress biomarkers [66]. Both mechanisms proposed are consistent with our results where only the two highest doses of THY were able to increase alpha-linolenic acid concentration, from 14 days of S on, while the three doses induced changes in all long chain-PUFA (LCPUFA), which began to be observed at 7 days of S.

In relation to the third hypothesis mentioned above, it is important to note that PUFA regulatory functions have gathered much interest recently [67,83–87]. Of great significance to us are the findings showing that docosahexaenoic and eicopentaenoic acids reduce ROS generation and increase glutathione, glutamate–cysteine ligase and glutathione peroxidase 4 levels in astrocytes in a dose-dependent manner (under basal condition and in H_2_O_2_-treated) [68]. Additionally, both n-3 PUFA activated transcription factor Nrf2 also in a dose-dependent manner. Thus, THY could initially promote increased circulating levels of PUFA but thereafter also PUFA could be contributing to generate an improved antioxidant status preventing its own peroxidation. This would explain the changes in FA that continue to occur even when there is no change in the circulating THY concentration or in the pS period, when the supply of this compound has been withdrawn. It would be of great importance to investigate the feasibility of this mechanism after THY supplementation in vivo, since the transcription factor Nrf2 could become a therapeutic target with beneficial effects in animal physiology [88].

In our study, since females were fully grown adults in their egg-laying peak, it was expected that performance traits remained optimal in all treatments [45,46,89,90]. Other previous studies have reported that mixtures with THY had positive effects on growth performance, egg production and egg physical characteristics depending on the dose [4,13,91], which could be due to differences in the experimental protocols used, especially regarding the age of the animals evaluated. Moreover, it has also been suggested that the effects of THY may be greatly dependent on the diet formulation (affecting digestibility of nutritional components and EOs) and the environment (challenging conditions such as the firsts weeks of growing, heat stress, etc.) thus, under optimal conditions, no further performance enhancement could be evidenced [92,93]. Also, we considered positive that THY supplementation did not affect physical egg traits nor the general well-being and histopathological features of the liver. In this regard, it is noteworthy to mention that steatosis or fatty degeneration observed in all livers is consistent with the reproductive status of the females (peak of laying) [94,95]. We ruled out that it is due to overfeeding, given daily feed intakes were within the normal range for the species [45,78] and that the composition of the feeds were in accordance with the recommendation for animals of this age and physiological state [42].

## Conclusion

This is the first study that using pure THY encompasses the in vivo effects of increasing concentrations of THY during and in the mid-term after chronic supplementation, the bioavailability of THY in the yolk and droppings, the total FA composition of the yolk and a series of indicators of productivity and general welfare status in adult quail. With this approach we showed dose and time dependent effects, that are consistent with THY direct and indirect antioxidant activities as well as its influence on other metabolic pathways. Thus, supplementation with THY4 and THY6 promotes the production of eggs with an improved nutritional quality, whose beneficial effects could be sustained for at least 21 days after a 28-day S period is finished. Thymol supplementation would be advisable for the production of healthier table eggs, and fertile eggs. Taken together with previous reports [15], it could also contribute to successful embryo development. Finally, the doses tested herein do not negatively affect productive performance traits nor liver histopathology.

## Supporting information

S1 Table Nutrient composition of administered diets. (DOC)

S2 Table Vitaminic premix composition of administered diets. (DOC)

S3 Table Concentration of feed total fatty acids. (DOC)

S4 Table Thymol concentration in supplemented feed and percentage incorporated into feed. (DOC)

S5 Table Percentage of thymol transferred to egg yolk and droppings in relation to the amount ingested. (DOC)

## References

[1] Lee KW, Everts H, Beynen AC. Essential oils in broiler nutrition. Int J Poult Sci. 2004;3:738–52.

[2] Brenes A, Roura E. Essential oils in poultry nutrition: Main effects and modes of action. Anim Feed Sci Technol. 2010;158(1–2):1–14.

[3] Madhupriya V, Shamsudeen P, Manohar GR, Senthilkumar S. Phyto feed additives in poultry nutrition–A review. Int J Sci Environ Technol. 2018;7(3):815–22.

[4] Radwan Nadia L, Hassan RA, Qota EM, Fayek HM. Effect of Natural Antioxidant on Oxidative Stability of Eggs and Productive and Reproductive Performance of Laying Hens. Int J Poult Sci. 2008;7(2):134–50.

[5] Abd El-Hack M, Alagawany M, Farag M, Tiwari R, Karthik K, Dhama K, et al Beneficial impacts of thymol essential oil on health and production of animals, fish and poultry: a review. J Essent Oil Res. 2016;28(5):365–82.

[6] Nagoor Meeran MF, Javed H, Al Taee H, Azimullah S, Ojha SK. Pharmacological Properties and Molecular Mechanisms of Thymol: Prospects for Its Therapeutic Potential and Pharmaceutical Development. Front Pharmacol. 2017;8:380 10.3389/fphar.2017.00380 28694777 PMC5483461

[7] Luna A, Tarifa F, Fernandez M., Caliva MJ, Pellegrini S, Zygadlo JA, et al Thymol, alpha tocopherol, and ascorbyl palmitate supplementation as growth enhancers for broiler chickens. Poult Sci. 2018; 98(2):1012–1016. 10.3382/ps/pey362 30165460

[8] Youdim KA, Deans SG. Dietary supplementation of thyme (*Thymus vulgaris L.*) essential oil during the lifetime of the rat: its effects on the antioxidant status in liver, kidney and heart tissues. Mech Ageing Dev. 1999;109(3):163–75. 10576332 10.1016/s0047-6374(99)00033-0

[9] Luna A, Labaque M, Zygadlo J, Marin R. Use of natural phenols as feed supplements with antioxidant effects on poultry products. Congr World Vet Poult Assoc. 2013.

[10] Omonijo FA, Ni L, Gong J, Wang Q, Lahaye L, Yang C. Essential oils as alternatives to antibiotics in swine production. Anim Nutr. 2018;4(2):126–36. 10.1016/j.aninu.2017.09.001 30140752 PMC6104524

[11] Botsoglou N., Christaki E, Fletouris DJ, Florou-Paneri P, Spais AB. The effect of dietary oregano essential oil on lipid oxidation in raw and cooked chicken during refrigerated storage. Meat Sci. 2002;62:259–65. 22061420 10.1016/s0309-1740(01)00256-x

[12] Luna A, Labaque MC, Zygadlo JA, Marin RH. Effects of thymol and carvacrol feed supplementation on lipid oxidation in broiler meat. Poult Sci. 2010;89(2):366–70. 10.3382/ps.2009-00130 20075292

[13] Hashemipour H, Kermanshahi H, Golian A, Veldkamp T. Effect of thymol and carvacrol feed supplementation on performance, antioxidant enzyme activities, fatty acid composition, digestive enzyme activities, and immune response in broiler chickens. Poult Sci. 2013;92(8):2059–69. 10.3382/ps.2012-02685 23873553

[14] Botsoglou N, Yannakopoulos AL, Fletouris DJ, Tserveni-Goussi AS, Fortomaris PD. Effect of dietary thyme on oxidative stability of egg yolk. J Agric Food Chem. 1997;45(10):3711–6.

[15] Luna A, Dambolena JS, Zygadlo JA, Marin RH, Labaque MC. Effects of thymol and isoeugenol feed supplementation on quail adult performance, egg characteristics and hatching success. Br Poult Sci. 2012;53(5):631–9. 10.1080/00071668.2012.721536 23281757

[16] Fernandez ME, Marin RH, Luna A, Zunino MP, Labaque MC. Thymol feed supplementation in quail alters the percentages of nutritionally relevant egg yolk fatty acids: effects throughout incubation. J Sci Food Agric. 2017;97(15):5233–40. 10.1002/jsfa.8407 28474397

[17] Simopoulos AP. The Importance of the Omega-6/Omega-3 Fatty Acid Ratio in Cardiovascular Disease and Other Chronic Diseases. Exp Biol Med. 2008;233(6):674–88. 10.3181/0711-MR-311 18408140

[18] Palmquist DL. Omega-3 Fatty Acids in Metabolism, Health, and Nutrition and for Modified Animal Product Foods. Prof Anim Sci. 2009;25(3):207–49.

[19] Surai PF, Sparks NHC. Comparative evaluation of the effect of two maternal diets on fatty acids, vitamin E and carotenoids in the chick embryo. Br Poult Sci. 2001;42(2):252–9. 10.1080/00071660120048519 11421335

[20] Cherian G. Nutrition and metabolism in poultry: Role of lipids in early diet. J Anim Sci Biotechnol. 2015;6(1). 10.1186/s40104-015-0029-9 PMC4487977 26137219

[21] Biesalski H, Dragsted L, Elmadfa I, Grossklaus R, Müller M, Schrenk D, et al Bioactive compounds: Definition and assessment of activity. Nutrition. 2009;25(11):1202–5. 19695833 10.1016/j.nut.2009.04.023

[22] Cross DE, Svoboda K, McDevitt RM, Acamovic T. The performance of chickens fed diets with and without thyme oil and enzymes. Br Poult Sci. 2003;44(sup1):18–9.

[23] Giannenas I, Koidis A, Botsoglou E, Dotas V, Mitsopoulos I, Florou-Paneri P, et al Hen Performance and Egg Quality as Affected by Dietary Oregano Essential Oil and alpha-tocopheryl Acetate Supplementation. Int J Poult Sci. 2005;4(7):449–54.

[24] Jang IS, Ko YH, Kang SY, Lee CY. Effect of a commercial essential oil on growth performance, digestive enzyme activity and intestinal microflora population in broiler chickens. Anim Feed Sci Technol. 2007;134(3–4):304–15.

[25] Hoffman-Pennesi D, Wu C. The effect of thymol and thyme oil feed supplementation on growth performance, serum antioxidant levels, and cecal Salmonella population in broilers. J Appl Poult Res. 2010;19(4):432–43.

[26] Ding X, Yu Y, Su Z, Zhang K. Effects of essential oils on performance, egg quality, nutrient digestibility and yolk fatty acid profile in laying hens. Anim Nutr. 2017;3(2):127–31. 10.1016/j.aninu.2017.03.005 29767138 PMC5941116

[27] Fairweather-Tait SJ. The concept of bioavailability as it relates to iron nutrition. Nutr Res. 1987;7(3):319–25.

[28] Bravo L. Polyphenols: Chemistry, Dietary Sources, Metabolism, and Nutritional Significance. Nutr Rev. 1998;56(11):317–33. 10.1111/j.1753-4887.1998.tb01670.x 9838798

[29] Takada M, Agata I, Sakamoto M, Yagi N, Hayashi N. On the metabolic detoxication of thymol in rabbit and man. J Toxicol Sci. 1979;4:341–50. 548583 10.2131/jts.4.341

[30] Austgulen LT, Solheim E, Scheline RR. Metabolism in rats of p-cymene derivatives: carvacrol and thymol. Pharmacol Toxicol. 1987;61(2):98–102. 2959918 10.1111/j.1600-0773.1987.tb01783.x

[31] Kohlert C, Van Rensen I, Marz R, Schindler G, Graefe EU, Veit M. Bioavailability and pharmacokinetics of natural volatile terpenes in animals and humans. Planta Med. 2000;66(6):495–505. 10.1055/s-2000-8616 10985073

[32] Kohlert C, Schindler G, März RW, Abel G, Brinkhaus B, Derendorf H, et al Systemic availability and pharmacokinetics of thymol in humans. J Clin Pharmacol. 2002;42(7):731–7. 12092740 10.1177/009127002401102678

[33] Thalhamer B, Buchberger W, Waser M. Identification of thymol phase I metabolites in human urine by headspace sorptive extraction combined with thermal desorption and gas chromatography mass spectrometry. J Pharm Biomed Anal. 2011;56(1):64–9. 10.1016/j.jpba.2011.04.014 21620603

[34] Haselmeyer A, Zentek J, Chizzola R. Effects of thyme as a feed additive in broiler chickens on thymol in gut contents, blood plasma, liver and muscle. J Sci Food Agric. 2015;95(3):504–8. 10.1002/jsfa.6758 24862829

[35] Oceľová V, Chizzola R, Pisarcikova J, Novak J, Ivanišinová O, Faix Š, et al Effect of Thyme Essential Oil Supplementation on Thymol Content in Blood Plasma, Liver, Kidney and Muscle in Broiler Chickens. Nat Prod Commun. 2016;11(10):1545–1550 30549619

[36] Pisarčíková J, Oceľová V, Faix Š, Plachá I, Calderón AI. Identification and quantification of thymol metabolites in plasma, liver and duodenal wall of broiler chickens using UHPLC-ESI-QTOF-MS. Biomed Chromatogr. 2016;31(5):e3881. 10.1002/bmc.3881 27808421

[37] Fernandez ME, Palacio MA, Labaque MC. Thymol detection and quantitation by solid-phase microextraction in faeces and egg yolk of Japanese quail. J Chromatogr B. 2017;1044–1045:39–46. 10.1016/j.jchromb.2016.12.042 28076773

[38] Krause EL, Ternes W. Bioavailability of the antioxidative thyme compounds thymol and p-cymene-2,3-diol in eggs. Eur Food Res Technol. 1999;209(2):140–4.

[39] EFSA. Scientific Opinion on safety and efficacy of CRINA® Poultry Plus (preparation of benzoic acid and essential oil compounds) as feed additive for chickens for fattening. EFSA J. 2012;10(3):2620–2642. 10.2903/j.efsa.2012.2620 PMC13151784 42112013

[40] National Institue of Health. Guide. Vol. Edition, E, Guide for the Care and Use of Laboratory Animals. 2011.

[41] Labaque MC, Kembro JM, Luna A, Marin RH. Effects of thymol feed supplementation on female Japanese quail (*Coturnix coturnix*) behavioral fear response. Anim Feed Sci Technol. 2013;183(1–2):67–72.

[42] National Research Council. Nutrient Requirements of Poultry: Ninth Revised Edition. The National Academies Press 1994.

[43] Folch J, Less M, Sloane Stanley G. A Simple Method for the Isolation and Purification of Total Lipides from Animal Tissues. J Biol Chem. 1957;226(1):497–509. 13428781

[44] Labaque MC, Martella M, Maestri D, Navarro J. The influence of diet composition on egg and chick traits in captive Greater Rhea females. Br Poult Sci. 2013;54(3):374–80. 10.1080/00071668.2013.791965 23796119

[45] Vercese F, Garcia EA, Sartori JR, Silva A de P, Faitarone ABG, Berto DA, et al Performance and egg quality of Japanese quails submitted to cyclic heat stress. Brazilian J Poult Sci. 2012;14:37–41.

[46] Vali N, Mottaghi S. The effect of using different levels of cinnamon and thyme powder on egg characteristics and fatty acids profile in japanese quail. CIBTech J Zool. 2016;5(3):2319–3883.

[47] Nikolova N, Kocevski D. Forming egg shape index as influenced by ambiente temperatures and age of hens. Biotechnol Anim Husb. 2006;22(1–2):119–25.

[48] Pellegrini S, Condat L, Marin R, Guzman D. Can Japanese quail male aggressions toward a female cagemate predict aggressiveness toward unknown conspecifics? Livest Sci 2019;222:65–70.

[49] Welfare Quality® consortium. Welfare Quality® Assessment protocol for poultry. 2009;76–98.

[50] Bernet D, Schmidt H, Meier W, Burkhardt-Holm P, Wahli T. Histopathology in fish: proposal for a protocol to assess aquatic pollution. J Fish Dis. 2001;22(1):25–34.

[51] Rautenberg GE, Amé MV, Monferrán MV, Bonansea RI, Hued AC. A multi-level approach using *Gambusia affinis* as a bioindicator of environmental pollution in the middle-lower basin of Suquía River. Ecol Indic. 2015;48:706–20.

[52] Di Rienzo J, Casanoves F, Balzarini M, Gonzalez L, Tablada M, Robledo CW. InfoStat. Versión 20. Córdoba, Argentina: Universidad Nacional de Córdoba; 2018.

[53] Aeschbach R, Loliger J, Scott B, Murcia A, Butler J, Halliwell M, et al Antioxidant actions of thymol, carvacrol, 6-gingerol, zingerone and hydroxytyrosol. Food Chem Toxicol. 1994;32(1):31–6. 7510659 10.1016/0278-6915(84)90033-4

[54] Nagoor Meeran M, Prince P. Protective effects of thymol on altered plasma lipid peroxidation and nonenzymic antioxidants in isoproterenol- induced myocardial infarcted rats. J Biochem Mol Toxicol. 2012;26(9):368–373. 10.1002/jbt.21431 22890907

[55] Horvathova E, Navarova J, Galova E, Sevcovicova A, Chodakova L, Snahnicanova Z, et al Assessment of Antioxidative, Chelating, and DNA-Protective Effects of Selected Essential Oil Components (Eugenol, Carvacrol, Thymol, Borneol, Eucalyptol) of Plants and Intact *Rosmarinus officinalis* Oil. J Agric Food Chem. 2014;62(28):6632–9. 10.1021/jf501006y 24955655

[56] Yu Y, Chao T, Chang W, Chang M, Lee M. Thymol reduces oxidative stress, aortic intimal thickening, and inflammation-related gene expression in hyperlipidemic rabbits. J Food Drug Anal. 2016;24(3):556–63. 10.1016/j.jfda.2016.02.004 28911561 PMC9336656

[57] Papageorgiou G, Botsoglou N, Govaris A, Giannenas I, Iliadis S, Botsoglou E. Effect of dietary oregano oil and alpha-tocopheryl acetate supplementation on iron-induced lipid oxidation of turkey breast, thigh, liver and heart tissues. J Anim Physiol Anim Nutr 2008;87(9–10):324–35. 10.1046/j.1439-0396.2003.00441.x 14507415

[58] Zou Y, Wang J, Peng J, Wei H. Oregano Essential Oil Induces SOD1 and GSH Expression through Nrf2 Activation and Alleviates Hydrogen Peroxide-Induced Oxidative Damage in IPEC-J2 Cells. Oxid Med Cell Longev. 2016;Article ID 5987183:13 pages. 10.1155/2016/5987183 PMC5220500 28105249

[59] Qin S, Hou DX. The Biofunctions of Phytochemicals and Their Applications in Farm Animals: The Nrf2/Keap1 System as a Target. Engineering. 2017;3(5):738–52.

[60] Surai K, Surai P, Speake B, Sparks N. Antioxidant-Prooxidant balance in the intestine: food for thought 2. Antioxidants. Curr Top Nutraceutical Res. 2004;2(1):27–46.

[61] Song P, Zhang R, Wang X, He P, Tan L, Ma X. Dietary grape-seed procyanidins decreased postweaning diarrhea by modulating intestinal permeability and suppressing oxidative stress in rats. J Agric Food Chem. 2011;59(11):6227–32. 10.1021/jf200120y 21534629

[62] Han M, Song P, Huang C, Rezaei A, Farrar S, Brown M, et al Dietary grape seed proanthocyanidins (GSPs) improve weaned intestinal microbiota and mucosal barrier using a piglet model. Oncotarget. 2016;7(49):80313–26. 10.18632/oncotarget.13450 27880936 PMC5348322

[63] Hayes JD, Ashford MLJ. Nrf2 orchestrates fuel partitioning for cell proliferation. Cell Metab. 2012;16(2):139–41. 10.1016/j.cmet.2012.07.009 22883227

[64] Souri H, Khatibjoo A, Taherpoor K, Hassan Abadi A, Fattahnia F, Askari M. Effect of *Thymus vulgaris* and *Satureja khuzestanica* Ethanolic Extracts on Broiler Chickens’ Performance and Immune Response. Iran J Appl Anim Sci. 2015;5(2):437–46.

[65] Choi J, Kim S, Yu R, Yun J. Monoterpene phenolic compound thymol promotes browning of 3T3-L1 adipocytes. Eur J Nutr. 2016;56(7):2329–41. 10.1007/s00394-016-1273-2 27431894

[66] Valenzuela R, Echeverria F, Ortiz M, Rincón-Cervera MÁ, Espinosa A, Hernandez-Rodas MC, et al Hydroxytyrosol prevents reduction in liver activity of Δ-5 and Δ-6 desaturases, oxidative stress, and depletion in long chain polyunsaturated fatty acid content in different tissues of high-fat diet fed mice. Lipids Health Dis. 2017;16(1):1–16. 10.1186/s12944-016-0392-3 28395666 PMC5387240

[67] Clarke SD, Jump DB. Nutrition and Gene Expression. Chapter 10. Regulation of Hepatic Gene Expression by Dietary Fats: A Unique Role for Polyunsaturated Fatty Acids. First Edit. Berdanier C, editor. Taylor & Francis; 2017 591 pages.

[68] Zgórzyńska E, Dziedzic B, Gorzkiewicz A, Stulczewski D, Bielawska K, Su K-P, et al Omega-3 polyunsaturated fatty acids improve the antioxidative defense in rat astrocytes via an Nrf2-dependent mechanism. Pharmacol Reports. 2017;69(5):935–42. 10.1016/j.pharep.2017.04.009 28662394

[69] Halliwell B, Rafter J, Jenner A. Health promotion by flavonoids, tocopherols, tocotrienols, and other phenols: direct or indirect effects? Antioxidant or not? Am J Clin Nutr. 2005;81(1):268S–276S. 15640490 10.1093/ajcn/81.1.268S

[70] Surai PF. Polyphenol compounds in the chicken/animal diet: from the past to the future. J Anim Physiol Anim Nutr (Berl). 2014;98(1):19–31. 23527581 10.1111/jpn.12070

[71] Yanishlieva NV, Marinova EM, Gordon MH, Raneva VG. Antioxidant activity and mechanism of action of thymol and carvacrol in two lipid systems. Food Chem. 1999;64(1):59–66.

[72] Satooka H, Kubo I. Effects of Thymol on B16-F10 Melanoma Cells. J Agric Food Chem. 2012;60(10):2746–52. 10.1021/jf204525b 22352891

[73] Llana-Ruiz-Cabello M, Gutiérrez-Praena D, Puerto M, Pichardo S, Jos Á, Cameán AM. In vitro pro-oxidant/antioxidant role of carvacrol, thymol and their mixture in the intestinal Caco-2 cell line. Toxicol Vitr. 2015;29(4):647–56. 10.1016/j.tiv.2015.02.006 25708581

[74] Shen Q, Zhou W, Li H, Hu L, Mo H. ROS Involves the Fungicidal Actions of Thymol against Spores of *Aspergillus flavus* via the Induction of Nitric Oxide. PLoS One. 2016;11(5):1–14. 10.1371/journal.pone.0155647 PMC4872997 27196096

[75] Shen G, Xu C, Hu R, Jain MR, Nair S, Lin W, et al Comparison of (−)-Epigallocatechin-3-Gallate Elicited Liver and Small Intestine Gene Expression Profiles Between C57BL/6J Mice and C57BL/6J/Nrf2 (−/−) Mice. Pharm Res. 2005;22(11):1805–20. 10.1007/s11095-005-7546-8 16132347

[76] James KD, Forester SC, Lambert JD. Dietary pretreatment with green tea polyphenol, (−)-epigallocatechin-3-gallate reduces the bioavailability and hepatotoxicity of subsequent oral bolus doses of (−)-epigallocatechin-3-gallate. Food Chem Toxicol. 2015;76:103–8. 10.1016/j.fct.2014.12.009 25528115 PMC4383035

[77] Wang D, Wang Y, Wan X, Yang CS, Zhang J. Green tea polyphenol (−)-epigallocatechin-3-gallate triggered hepatotoxicity in mice: Responses of major antioxidant enzymes and the Nrf2 rescue pathway. Toxicol Appl Pharmacol. 2015;283(1):65–74. 10.1016/j.taap.2014.12.018 25585349

[78] Sahin K, Orhan C, Tuzcu M, Ali S, Sahin N, Hayirli A. Epigallocatechin-3-gallate prevents lipid peroxidation and enhances antioxidant defense system via modulating hepatic nuclear transcription factors in heat-stressed quails. Poult Sci. 2010;89(10):2251–8. 10.3382/ps.2010-00749 20852116

[79] Youdim KA, Deans SG. Effect of thyme oil and thymol dietary supplementation on the antioxidant status and fatty acid composition of the ageing rat brain. Br J Nutr. 2000;83(1):87–93. 10703468

[80] Lin CC, Wu SJ, Chang CH, Ng LT. Antioxidant activity of *Cinnamomum cassia*. Phyther Res. 2003;17(7):726–30. 10.1002/ptr.1190 12916067

[81] Nain S, Renema RA, Korver DR, Zuidhof MJ. Characterization of the n-3 polyunsaturated fatty acid enrichment in laying hens fed an extruded flax enrichment source. Poult Sci. 2012;91(7):1720–32. 10.3382/ps.2011-02048 22700520

[82] Jamroz D, Wiliczkiewicz A, Wertelecki T, Orda J, Skorupińska J. Use of active substances of plant origin in chicken diets based on maize and locally grown cereals. Br Poult Sci. 2005;46(4):485–93. 10.1080/00071660500191056 16268107

[83] Clarke S. Polyunsaturated Fatty Acid Regulation of Gene Transcription: A Molecular Mechanism to Improve the Metabolic Syndrome. J Nutr. 2001;131(4):1129–32. 10.1093/jn/131.4.1129 11285313

[84] Lukiw W, Cui J, Marcheselli V, Bodker M, Botkjaer A, Gotlinger K, et al A role for docosahexaenoic acid–derived neuroprotectin D1 in neural cell survival and Alzheimer disease. J Clin Invest. 2005;115(10):2774–83. 10.1172/JCI25420 16151530 PMC1199531

[85] Kawakita E, Hashimoto M, Shido O. Docosahexaenoic acid promotes neurogenesis in vitro and in vivo. Neuroscience. 2006;139(3):991–7. 10.1016/j.neuroscience.2006.01.021 16527422

[86] Little SJ, Lynch MA, Manku M, Nicolaou A. Docosahexaenoic acid-induced changes in phospholipids in cortex of young and aged rats: A lipidomic analysis. Prostaglandins, Leukot Essent Fat Acids. 2007;77(3):155–62. 10.1016/j.plefa.2007.08.009 17928211

[87] Cipollina C. Endogenous generation and signaling actions of omega-3 fatty acid electrophilic derivatives. Biomed Res Int. 2015;Article ID 501792:13 pages. 10.1155/2015/501792 PMC4538325 26339618

[88] Copple I, Dinkova-kostova A, Kensler T, Liby K, Wigley W. NRF2 as an Emerging Therapeutic Target. Oxid Med Cell Longev. 2017;Article ID 8165458:2 pages. 10.1155/2017/8165458 PMC5306997 28250892

[89] Nestor J, Bacon W, Velleman S, Anderson J, Patterson R. Effect of Selection for Increased Body Weight and Increased Plasma Yolk Precursor on Developmental Stability in Japanese Quail. Poult Sci. 2002;81(2):160–168. 10.1093/ps/81.2.160 11873824

[90] Alagawany M, Abd El-Hack ME, Farag MR, Elnesr SS, El-Kholy MS, Saadeldin IM, et al Dietary supplementation of Yucca schidigera extract enhances productive and reproductive performances, blood profile, immune function, and antioxidant status in laying Japanese quails exposed to lead in the diet. Poult Sci. 2018;97(9):3126–37. 10.3382/ps/pey186 29846703

[91] Cross D, McDevitt R, Hillman K, Acamovic T. The effect of herbs and their associated essential oils on performance, dietary digestibility and gutmicroflora in chickens from7 to 28 days of age. Br Poult Sci. 2007;48(4):496–506. 10.1080/00071660701463221 17701503

[92] Lee K, Everts H, Kappert H, Yeom K, Beynen A. Dietary carvacrol lowers body weight gain but improves feed conversion in female broiler chickens. J Appl Poult Res. 2003;12(4):394–399.

[93] Ocak N, Erener G, Burak A, Sungu M, Ozmen A. Performance of broilers fed diets supplemented with dry peppermint (Mentha piperita L.) or thyme (Thymus vulgaris L.) leaves as growth promoter source. Czech J Anim Sci 53:169–175 (2008). Czech J Anim Sci. 2008;53(4):169–75.

[94] Butler E. Fatty liver diseases in the domestic fowl-a review. Avian Pathol. 1976;5(1):1–14. 10.1080/03079457608418164 18777323

[95] Akiba Y, Jensen LS, Barb CR, Kraeling RR. Plasma Estradiol, Thyroid Hormones, and Liver Lipid Content in Laying Hens Fed Different Isocaloric Diets. J Nutr. 1982;112(2):299–308. 10.1093/jn/112.2.299 7057268

