# Supplementary material for: Dynamics of thymol dietary supplementation in quail (Coturnix japonica): Linking bioavailability, effects on egg yolk total fatty acids and performance traits
Source: PLoS One. 2019 May 9;14(5):e0216623. doi: 10.1371/journal.pone.0216623 (PMC6508865; doi:10.1371/journal.pone.0216623)
Supplement: S1 Table — (DOC) [file pone.0216623.s001.doc]

**S1 Table. Nutrient composition of administered diets**

| Nutrient | Content (g/kg diet) |
| --- | --- |
| Minimum crude protein | 200 |
| Minimum fat matter | 55 |
| Maximum crude fiber | 51.5 |
| Maximum total minerals | 75 |
| Maximum calcium | 29 |
| Minimum calcium | 25 |
| Maximum phosphorus | 8.5 |
| Minimum phosphorus | 7.5 |

(20% of Crude Protein and 2900kcal of Metabolizable Energy/kg diet; NRC Publications, Ninth Edition, 1994). Basal feed was commercially obtained from Marcelo E. Hoffmann e Hijos S.A. Thus, both THY-supplemented and control (basal and vehicle) layer diets had corn, disabled soybean, wheat bran, soybean pellets, sunflower pellets, calcium, salt, vitamins, minerals and phosphate in identical proportions.
