# Supplementary material for: Dynamics of thymol dietary supplementation in quail (Coturnix japonica): Linking bioavailability, effects on egg yolk total fatty acids and performance traits
Source: PLoS One. 2019 May 9;14(5):e0216623. doi: 10.1371/journal.pone.0216623 (PMC6508865; doi:10.1371/journal.pone.0216623)
Supplement: S2 Table — (DOC) [file pone.0216623.s002.doc]

**S2 Table. Vitaminic premix composition of administered diets (20kg/ton feed)**

| Component | Content |
| --- | --- |
| Folic acid | 15 mg |
| Niacin | 500 mg |
| Calcium pantothenate | 250 mg |
| Vitamin A | 400.000 UI |
| Vitamin D3 | 110.000 UI |
| Vitamin E | 250 UI |
| Vitamin B1 | 25 mg |
| Vitamin B2 | 200 mg |
| Vitamin B12 | 400 μg |
| Vitamin K3 | 100 mg |
| Antioxidant (BHT 12%; BHA 3,5%) | 3 g |
| Iodo | 30 mg |
| Manganese | 3.5 g |
| Zinc | 3 g |
| Copper | 250 mg |
| Iron | 1.5 g |
| Selenium | 5 mg |
| Phosphorus | 100 g |
| Phytase | 20000 u/g 788 mg |
| Methionine | 50 g |
| Choline | 7.8 g |
| Canthaxanthin | 2 mg |
| Aluminosilicate | 100 g |
| Excipient sufficient amount | 1000 g |
