# Supplementary material for: Dynamics of thymol dietary supplementation in quail (Coturnix japonica): Linking bioavailability, effects on egg yolk total fatty acids and performance traits
Source: PLoS One. 2019 May 9;14(5):e0216623. doi: 10.1371/journal.pone.0216623 (PMC6508865; doi:10.1371/journal.pone.0216623)
Supplement: S3 Table — (DOC) [file pone.0216623.s003.doc]

**S3 Table. Concentration of feed total fatty acids.***

| Fatty acid | Mean ± SEM (g/100g FAME) | | | p-Value |
| --- | --- | --- | --- | --- |
| 14:0 (Miristic) | 0.06 | ± | 0.00 | 0.38 |
| 16:0 (Palmitic) | 12.44 | ± | 0.06 | 0.85 |
| 16:1 (Palmitoleic) | 0.10 | ± | 0.00 | 0.11 |
| 18:0 (Stearic) | 4.90 | ± | 0.17 | 0.63 |
| 18:1 (Oleic) | 31.91 | ± | 0.10 | 0.53 |
| 18:2 (Linoleic) | 45.05 | ± | 0.32 | 0.89 |
| 18:3 (Linolenic) | 5.49 | ± | 0.03 | 0.62 |
| 20:4 (Arachidonic) | 0.05 | ± | 0.00 | 0.19 |
| SFA | 17.41 | ± | 0.21 | 0.65 |
| MUFA | 32.01 | ± | 0.10 | 0.51 |
| PUFA | 50.59 | ± | 0.29 | 0.89 |

Mean ± SEM.

FAME= Fatty acid Methyl Esters

SFA = saturated fatty acids (14:0 + 16:0 + 18:0).

MUFA = monounsaturated fatty acids (16:1 + 18:1)

PUFA = polyunsaturated fatty acids (18:2 + 18:3)

*Two control diets (basal and vehicle) and three supplemented with 2, 4, and 6.25g of thymol / kg of feed.

One-way ANOVA was used for data analysis. Since no statistical differences were found between the diets supplied in terms of total fatty acids content, the Table is only showing the overall averages (n=6 aliquots per diet were analyzed. N=30 aliquots total).
