# Supplementary material for: Dynamics of thymol dietary supplementation in quail (Coturnix japonica): Linking bioavailability, effects on egg yolk total fatty acids and performance traits
Source: PLoS One. 2019 May 9;14(5):e0216623. doi: 10.1371/journal.pone.0216623 (PMC6508865; doi:10.1371/journal.pone.0216623)
Supplement: S4 Table — (DOC) [file pone.0216623.s004.doc]

| **THY concentration (ng/g)** | | | | | | | | | **Percentage of THY incorporated to the feed (**‡**)** | | | | | | | | |
| --- | --- | --- | --- | --- | --- | --- | --- | --- | --- | --- | --- | --- | --- | --- | --- | --- | --- |
| **THY2** | | | **THY4** | | | **THY6** | | | **THY2** | | | **THY4** | | | **THY6** | | |
| 8770.84 | ± | 916.25 | 22924.45 | ± | 1784.21 | 36844.51 | ± | 4786.15 | 0.44 | ± | 0.05 | 0.57 | ± | 0.04 | 0.59 | ± | 0.08 |

**S4 Table. Thymol concentration in supplemented feed and percentage incorporated into feed.***

Mean ± SEM.

*Supplemented feed: doses 2, 4, 6.25g of thymol/kg of feed (THYMOL 2,4, and 6, respectively)

(‡) Percentage of THY incorporated to the feed = (ng of THY detected by HS-SPME per g of feed / ng of THY pulverized per g of feed) *100
