# Supplementary material for: Dynamics of thymol dietary supplementation in quail (Coturnix japonica): Linking bioavailability, effects on egg yolk total fatty acids and performance traits
Source: PLoS One. 2019 May 9;14(5):e0216623. doi: 10.1371/journal.pone.0216623 (PMC6508865; doi:10.1371/journal.pone.0216623)
Supplement: S5 Table — (DOC) [file pone.0216623.s005.doc]

**S5 Table. Percentage of thymol transferred to egg yolk and droppings in relation to the amount ingested.***

| **Matrix** |  | **THY concentration (ng/g)** | | | | | | | | | **Percentage of THY transferred (**‡**)** | | | | | | | | | |  |
| --- | --- | --- | --- | --- | --- | --- | --- | --- | --- | --- | --- | --- | --- | --- | --- | --- | --- | --- | --- | --- | --- |
|  |  | **THY2** | | | **THY4** | | | **THY6** | | | | **THY2** | | | **THY4** | | | **THY6** | | | |
| **Yolk** | 2 days of S | 12.49 | ± | 0.87 | 26.14 | ± | 1.39 | 42.92 | ± | 5.01 | | 0.02 | ± | 1.30E-03 | 0.01 | ± | 7.90E-04 | 0.02 | ± | 2.40E-03 | |
|  | 9 days of S | 155.78 | ± | 10.34 | 297.29 | ± | 20.14 | 406.93 | ± | 41.00 | | 0.23 | ± | 0.02 | 0.17 | ± | 0.01 | 0.14 | ± | 0.01 | |
|  | 16 days of S | 150.14 | ± | 6.23 | 294.9 | ± | 31.51 | 372.32 | ± | 46.32 | | 0.22 | ± | 0.01 | 0.17 | ± | 0.02 | 0.13 | ± | 0.02 | |
|  | 26 days of S | 169.13 | ± | 12.34 | 282.41 | ± | 25.99 | 377.15 | ± | 42.00 | | 0.25 | ± | 0.02 | 0.16 | ± | 0.01 | 0.13 | ± | 0.01 | |
|  | pS | 17.91 | ± | 2.14 | 36.32 | ± | 3.02 | 69.52 | ± | 4.25 | | 0.03 | ± | 3.20E-03 | 0.02 | ± | 1.70E-03 | 0.03 | ± | 2.40E-03 | |
|  |  |  |  |  |  |  |  |  |  |  | |  |  |  |  |  |  |  |  |  | |
| **Droppings** | 2 days of S | 51.75 | ± | 22.45 | 63.55 | ± | 13.42 | 84.98 | ± | 16.38 | | 0.53 | ± | 0.06 | 0.25 | ± | 0.05 | 0.21 | ± | 0.04 | |
|  | 9 days of S | 229.33 | ± | 16.22 | 459 | ± | 41.81 | 550 | ± | 25.53 | | 2.33 | ± | 0.14 | 1.78 | ± | 0.16 | 1.33 | ± | 0.06 | |
|  | 16 days of S | 237.43 | ± | 16.33 | 490.86 | ± | 32.8 | 720.99 | ± | 59.19 | | 2.41 | ± | 0.17 | 1.91 | ± | 0.13 | 1.74 | ± | 0.14 | |
|  | 26 days of S | 341.57 | ± | 24.31 | 541.8 | ± | 35.86 | 650.26 | ± | 56.37 | | 3.47 | ± | 0.25 | 2.11 | ± | 0.14 | 1.57 | ± | 0.14 | |
|  | pS | 4.37 | ± | 1.79 | 30.58 | ± | 2.44 | 37.95 | ± | 3.91 | | 0.04 | ± | 0.02 | 0.12 | ± | 0.01 | 0.09 | ± | 0.01 | |

Mean ± SEM.

*Females were fed diets with 2, 4, and 6.25g of thymol/kg (THYMOL 2,4 and 6, respectively) during 2, 9, 16, and 26 days of the supplementation period (S) and after 17 days of supplement withdrawal (pS).

(‡) Percentage of THY transferred = [(ng of THY detected per g of egg yolk or droppings x weight of egg yolk or daily deposited droppings) / (ng THY detected per g of feed * g of daily feed intake)] x 100

Average egg yolk weight = 3,94 g

Average daily deposition of fresh droppings = 27 g
